# Timing of hydrocortisone therapy in neonates with shock: a systematic review, meta-analysis, and clinical practice guideline

**DOI:** 10.3389/fped.2025.1491976

**Published:** 2025-03-12

**Authors:** Viraraghavan Vadakkencherry Ramaswamy, Gunjana Kumar, Abdul Kareem Pullattayil S, Abhishek S. Aradhya, Pradeep Suryawanshi, Mohit Sahni, Supreet Khurana, Shiv Sajan Saini, Ravishankar K, Shashi Kant Dhir, Deepak Chawla, Praveen Kumar, Kiran More

**Affiliations:** ^1^ Department of Neonatology, Ankura Hospital for Women and Children, Hyderabad, India; ^2^ Department of Neonatology, National Institute of Medical Sciences, Jaipur, India; ^3^ Queen's University Library, Queen's University, Kingston, ON, Canada; ^4^ Department of Neonatology, Ovum Women and Child Specialty Hospital, Bengaluru, India; ^5^ Department of Neonatology, Bharati Vidyapeeth Medical College Hospital, Pune, India; ^6^ Department of Neonatology, Nirmal Hospital, Surat, India; ^7^ Department of Neonatology, Government Medical College and Hospital, Chandigarh, India; ^8^ Department of Neonatology and Head, Postgraduate Institute of Medical Education and Research, Chandigarh, India; ^9^ Department of Neonatology, Sowmya Children’s Hospital, Hyderabad, India; ^10^ Department of Pediatrics, Guru Gobind Medical College, Faridkot, India; ^11^ Division of Neonatology, MRR Children’s Hospital, Mumbai, India

**Keywords:** neonates, hydrocortisone, shock, clinical practical guidelines, meta-analysis, systematic review

## Abstract

**Background:**

The effect of the timing of initiation of hydrocortisone in neonatal shock has not been evaluated. The objective of this systematic review was to compare the effect of earlier vs. later initiation of hydrocortisone in neonatal shock.

**Methods:**

Medline, Embase, and CENTRAL were searched from inception until 15 May 2024. Randomized controlled trials (RCTs) and non-RCTs were eligible for inclusion. A random effects meta-analysis was used to synthesize the data. The evidence certainty was evaluated according to Grading of Recommendations Assessment, Development, and Evaluation (GRADE). A clinical practice guideline was formulated as recommended by the GRADE group.

**Results:**

Of the 3,757 titles and abstracts screened, 20 studies were included: 7 RCTs and 13 non-RCTs. While clinical benefit or harm could not be ruled out for the outcome of mortality from the meta-analysis of the RCTs [early initiation risk ratio (RR): 0.46, 95% confidence interval (CI): 0.03–7.92; late initiation RR: 0.43, 95% CI: 0.12–1.47], the non-RCTs included in the narrative review suggested that late hydrocortisone initiation might be associated with increased risk of mortality. The meta-analysis indicated that early and late hydrocortisone administration may be associated with an increased response to treatment therapy (early initiation RR: 1.85, 95% CI: 1.26–2.71; late initiation RR: 2.50, 95% CI: 1.16–5.39). Late hydrocortisone initiation may increase the risk of necrotizing enterocolitis (NEC) ≥ stage 2 (RR: 2.46, 95% CI: 1.19–5.08). The evidence certainty was very low for most of the outcomes evaluated.

**Conclusion:**

The early use of hydrocortisone in neonates with shock requiring vasopressors is associated with better outcomes and no major adverse effects. Later institution of hydrocortisone therapy in neonatal shock may improve the response to therapy but may be associated with adverse outcomes including mortality and NEC. The results are to be interpreted with caution as the evidence certainty was predominantly very low.

**Systematic Review Registration:**

https://www.crd.york.ac.uk/PROSPERO/view/CRD42023432169, identifier: CRD42023432169.

## 1 Introduction

The use of hydrocortisone (HC) as an adjunct to inotropes in the treatment of neonatal shock is increasing, especially in extremely low birth weight (ELBW) neonates (1). Hydrocortisone administration in vasopressor-resistant shock has been shown to be associated with improved blood pressure (BP) in neonatal shock (2). The American Academy of Pediatrics (2022) concluded that prophylactic early use of hydrocortisone (≤7 days) is associated with a decreased risk of mortality or bronchopulmonary dysplasia (BPD) in ELBW infants exposed to chorioamnionitis (3). Similarly, the Canadian Paediatric Society 2020 recommended the use of early hydrocortisone (within 24–48 h) in extremely low gestational age neonates (ELGANs) born at <28 weeks’ gestation or those born through chorioamnionitis (3). There are no specific guidelines for hydrocortisone usage in vasopressor-resistant shock in neonates, especially with relation to its timing of initiation and dosage.

The mechanism of action of hydrocortisone in neonates with vasopressor-resistant shock includes sensitization of the cardiovascular system to catecholamines through the upregulation of adrenergic receptors, inhibition of catecholamine metabolism, and stabilization of capillary integrity (4). Hydrocortisone also facilitates the reuptake of norepinephrine into the sympathetic system and the expression of angiotensin type 2 receptors in the myocardium, thereby stabilizing the cardiovascular system and maintaining its sensitivity to vasopressors (4).

The etiology of adrenal insufficiency differs between term and preterm neonates (2, 5). While very preterm neonates are known to have primary adrenal insufficiency, the etiopathogenesis in both preterm and term neonates with shock could also be attributed to relative adrenal insufficiency (RAI) (6, 7). RAI is presumed to be due to hypothalamic-pituitary-adrenal (HPA) axis dysfunction (8). In a neonate with shock, the HPA axis may be affected due to reasons such as decreased secretion of cortisol from the adrenal gland secondary to ischemia, resistance of adrenal receptors to adrenocorticotrophic hormone (ACTH) due to inflammatory cytokines, and, finally, a lack of corticosteroid reserve in the adrenal gland relative to the increased metabolic demands (2, 4, 9). Unlike in pediatric and adult populations where there are specific cut-off levels to define primary and RAI, the definitions in neonates are contentious (8, 10, 11). Furthermore, studies in neonates with shock have shown conflicting results for the correlation between baseline serum cortisol levels and treatment response of shock or other adverse events with hydrocortisone administration guided by cortisol levels (12–16).

There is a lacuna in the literature as to when hydrocortisone has to be considered in neonates with shock. Hence, this systematic review and meta-analysis was conducted specifically to evaluate the timing of hydrocortisone initiation in relation to vasopressor therapy. A scoping review of the literature suggested that there was no randomized controlled trial (RCT) comparing hydrocortisone administration with respect to the timing of hydrocortisone initiation in relation to vasopressor therapy in neonates with shock. Hence, we conducted a systematic review and meta-analysis of studies comparing hydrocortisone therapy vs. no hydrocortisone therapy and addressed our objective through sub-group analyses.

## 2 Methods

### 2.1 Inclusion criteria

*Population (P):* Preterm and term neonates (of ≤28 days) diagnosed with shock (as defined by the authors) who were treated with volume expansion and/or inotropes.

*Intervention (I):* Hydrocortisone of any dosage along with the initiation of the first inotrope (dopamine, dobutamine, epinephrine, or vasopressin). Early vs. late initiation of hydrocortisone was defined as follows:

*Early:* Initiation of hydrocortisone prior to the initiation of inotropes or along with the addition of the first inotrope or with increasing requirement of inotropes [a cut-off vasoactive-inotropic score (VIS) of ≤10 was taken]. VIS is calculated as follows:

VIS = 1 × Dopamine (μg/kg/min) + 1 × Dobutamine (μg/kg/min) + 100 × Epinephrine (μg/kg/min) + 100 × Norepinephrine (μg/kg/min) + 10 × Milrinone (μg/kg/min) + 10,000 × Vasopressin (IU/kg/min).

*Late:* Initiation of hydrocortisone when high dosages of inotropes were required (VIS of > 10).

*Comparator (C):* Use of inotropes alone.

*Outcomes (O):* The primary outcomes were mortality and response to therapy (as defined by the authors) between 6 and 24 hours after hydrocortisone initiation. Secondary outcomes included blood culture-positive sepsis, necrotizing enterocolitis (NEC) ≥ stage 2 (17), major brain injury (MBI) [intraventricular hemorrhage (IVH) > grade 2 and/or cystic periventricular leukomalacia (PVL)] (18, 19), retinopathy of prematurity (ROP) requiring intervention (20), BPD [defined as oxygen requirement at 36 weeks’ post-menstrual age (PMA)], patent ductus arteriosus (PDA) requiring intervention (medical or surgical), change in BP, duration of inotropes, invasive mechanical ventilation, and hospitalization.

*Study designs (S):* RCTs; observational studies of either prospective, retrospective, or pre-post design; and conference abstracts were eligible for inclusion. There were no language restrictions. Descriptive reviews and systematic reviews were excluded.

*Time frame (T):* From inception of the searched databases until 15 May 2024.

Medline, Embase, and CENTRAL were searched from their inception until 15 May 2024 (Supplementary Table S1). Title and abstract screening for the inclusion of studies in the systematic review was performed using online software (Covidence, Veritas Health Innovation, Melbourne, Australia) by two authors blinded to each other (21). Discrepancies were resolved by consensus. The reference lists of the included studies and other similar systematic reviews were searched for potentially eligible studies.

### 2.2 Data extraction and synthesis

Two authors extracted data using a pre-specified pro forma. Data synthesis was performed using R software version 3.6.2 (R Foundation for Statistical Computing, Vienna, Austria) (22). A random effects pair-wise meta-analysis was performed using the Mantel–Haenszel method for binary outcomes, and the inverse variance method for continuous outcomes.

### 2.3 Risk of bias assessment

The risk of bias assessment was performed using the Cochrane risk of bias tool version 2.0 for RCTs (23) and Risk Of Bias in Non-randomized Studies of Interventions (ROBINS-I) for non-RCTs (24) by two authors independently. Disagreements were resolved by consensus.

### 2.4 Certainty of evidence assessment

Evidence certainty was assessed as per the Grading of Recommendations Assessment, Development, and Evaluation (GRADE) guidelines (25). The results of the systematic review and meta-analysis were reported according to modified GRADE recommendations (26, 27) (Supplementary Table S2).

### 2.5 Evidence to Decision framework and formulation of recommendations

The Evidence to Decision (EtD) framework according to the GRADE working group guidelines was used to arrive at recommendations (28).

### 2.6 Ethical approval

For this study design, i.e., a systematic review, meta-analysis, and clinical practice guideline, ethical approval is not required.

## 3 Results

**Figure 1:**
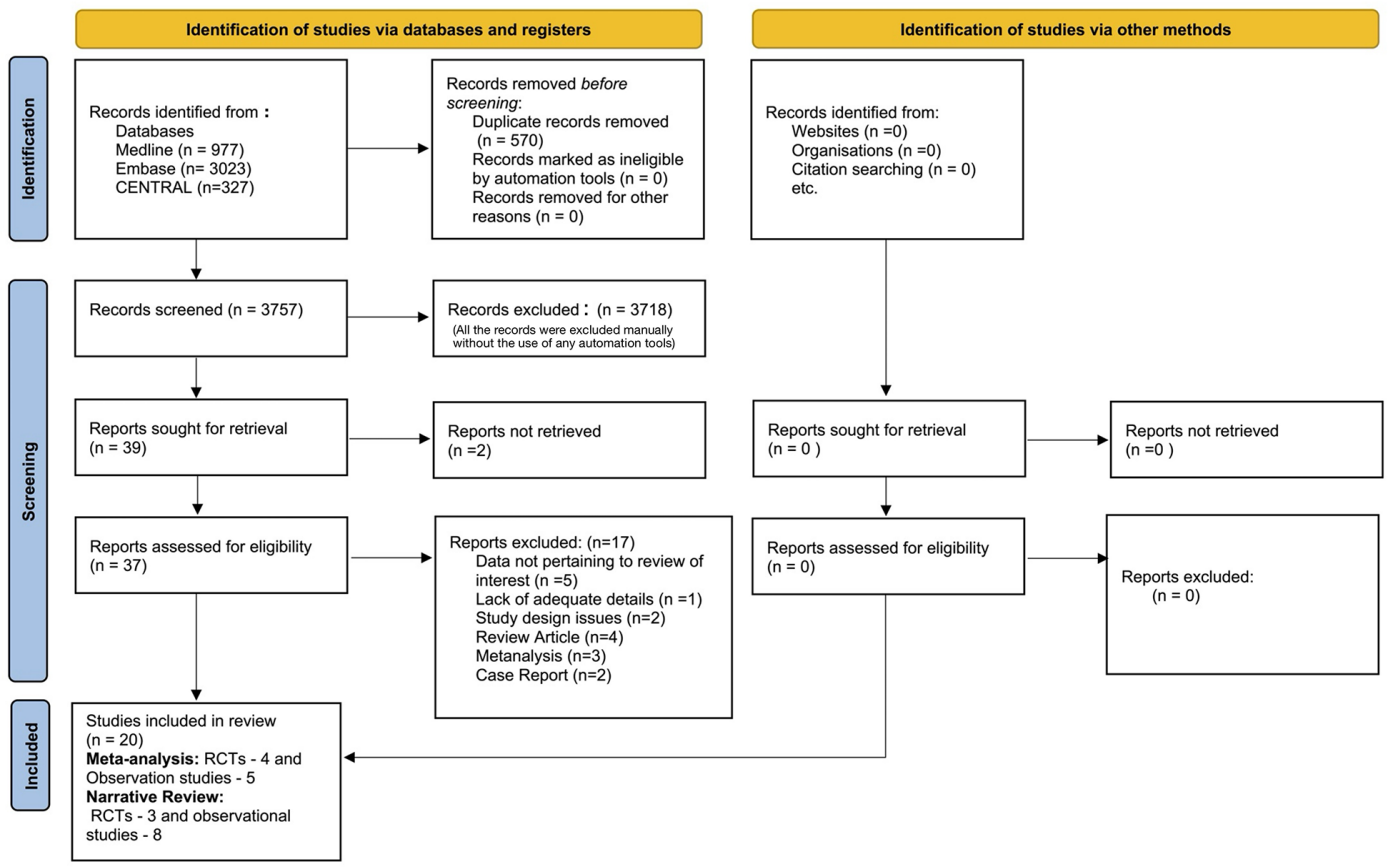
PRISMA diagram of the literature search.

Of the 3,757 titles and abstracts screened, 20 studies were included: 7 RCTs and 13 observational studies (1, 4, 12–15, 29–42). Of these, four RCTs (15, 33, 35, 39) and five observational studies (4, 29, 36, 37, 42) were synthesized in a meta-analysis; three RCTs (14, 31, 34) and eight observational studies (1, 12, 13, 30, 32, 38, 40, 41) were described in the narrative review. Amongst the observational studies, only those that had compared neonates with similar baseline characteristics and those that had adjusted for baseline sickness were included in the data synthesis, and the rest were included in the narrative review. The PRISMA flow is provided in Figure 1. The characteristics of the studies included in the meta-analyses are given in Tables 1, 2 and those included in the narrative review are provided in Table 3.

**Table 1:** Characteristics of the randomized controlled trials included in the meta-analysis.

Author, Year, Country	Intervention (number of participants)	Comparator (number of participants)	GA (weeks) mean ± SD or median (range)	BW (g) mean ± SD or median (range)	Time of initiation of hydrocortisone	Dose and duration of hydrocortisone	Other comments
Hochwald, 2013, Canada	11	11	I: 26.1 ± 1.5 C: 25.6 ± 1.4	I: 870 (555–1,120) C: 817 (585–1,030)	Early (HC started along with the administration of the first inotrope)	Initial dose of 2 mg/kg, 6 h after 1 mg/kg q 6 h for three doses, followed by 0.5 mg/kg q 6 h for four doses	- ≤30-week-old infants with ≤ 1,250 g BW within 48 h of postnatal age were enrolled - Definition of hypotension: MAP < GA - IBP monitoring was done - Both groups received two doses of fluid boluses (10 ml/kg) prior to randomization - The initial dose of dopamine used was 5 μg/kg/min and then titrated. If needed, an epinephrine infusion at 0.05 μg/kg/min was added and titrated
Kovacs, 2019, USA	16	19	I: 39 (38–40) C: 39 (38–40)	I: 3,625 (3,390–3,663) C: 3,500 (3,250–3,850)	Early (HC started along with the administration of the first inotrope)	0.5 mg/kg/d q 6 h until the re-warming period in neonates undergoing therapeutic hypothermia	- Infants were enrolled at 36 weeks who were undergoing therapeutic hypothermia for HIE - Definition of hypotension: MAP < GA - IBP monitoring was done - Both groups received two doses of fluid boluses (10 ml/kg over 15 min) prior to randomization - The dose of dopamine used was 6–20 μg/kg/min in both groups. Dobutamine was added if there was myocardial dysfunction. Inotropes were tapered at the clinicians’ discretion - Baseline cortisol levels were low in both groups and the values decreased in the placebo group - The HC group had a statistically significant decreased risk of electrographic seizures, but the requirement for anti-epileptic drug therapy and abnormal MRI were similar in both groups - The cumulative and maximum dose of inotrope therapy was significantly lower in the HC group - The regression modeling predicted HC to be associated with a 2.2 mm Hg increase in pulse pressure
Ng, 2006, Hong Kong	24	24	I: 27.2 (25.4–29.1) C: 26.0 (25.2–29.9)	I: 918 (729–1,223) C: 920 (648–1,189)	Early (HC was initiated if the dopamine requirement was ≥10 μg/kg/min)	1 mg/kg q 8 h for 5 days	- <32 weeks and <1,500 g infants within 7 days of life were enrolled - Definition of hypotension: MAP < GA - IBP monitoring was done - Both groups received fluid boluses (30 ml/kg) prior to randomization - HC was initiated if the dopamine requirement was ≥10 μg/kg/min. When 20 μg/kg/min dopamine and dobutamine failed to maintain MAP, an epinephrine infusion at a rate of 0.2 μg/kg/min was started. The dose was increased at a rate of 0.2 μg/kg/min q 30 min until the BP normalized - Serum cortisol level prior to randomization was comparable between the groups - Glycosuria was significantly higher in the HC group
Salas, 2014, Argentina	25	25	I: 38 ± 1.4 C: 38 ± 1.8	I: 2,936 ± 680 C: 3,184 ± 805	Late (HC was started at dopamine ≥14 μg/kg/min and/or after starting epinephrine)	2.5 mg/kg q 12 h for 48 h	- ≥37-week-old infants of any postnatal age were included - Definition of hypotension: MAP ≤ 5th centile - Both IBP and NIBP monitoring was done - Both groups received 10–20 ml/kg of volume expansion - Dopamine was started at 5–10 μg/kg/min and gradually increased. Dobutamine was used in neonates with poor cardiac contractility. If hypotension was persistent, epinephrine at 0.1 μg/kg/min was started - This was an open trial and six out of the 25 patients in placebo group received HC - Hyperglycemia was significantly higher in those infants who received epinephrine and HC (50%) - Though not statistically significant, two neonates in the HC group developed hypertension compared to none in the placebo group

GA, gestational age; BW, birth weight; I, intervention group; C, control group; SD, standard deviation; q, every; HC, hydrocortisone; IVH, intraventricular hemorrhage; BPD, bronchopulmonary dysplasia; BP, blood pressure; MAP, mean arterial pressure; IBP, invasive BP; NIBP, non-invasive BP; VLBW, very low birth weight. HIE, hypoxic-ischemic encephalopathy; NI, no information.

**Table 2:** Characteristics of the observational studies included in the meta-analysis

Author, Year, Country, Study design	Intervention (number of participants)	Comparator (number of participants)	GA (w) Mean ± SD or Median (range)	BW (g) Mean ± SD or Median (range)	Time of initiation of hydrocortisone	Dose and duration of hydrocortisone	Other comments
Altit, 2018, Canada, retrospective study	39	23	I: 26 ± 1.9 C: 27.1 ± 2.2	I: 828 ± 339 C: 1,013 ± 485	Late (mean VAI score in HC group was 15)	There was no uniform protocol for HC initiation. The cumulative dose of HC received was 10.5 mg/kg	- Preterm neonates of ≤34 weeks with culture-proven sepsis and/or NEC were included - Definition of hypotension was not reported - Both IBP and NIBP were used - Both groups received volume expansion prior to HC initiation - Dose of inotropes and the titration were clinician-dependent - The HC group was sicker compared to the no HC group - VAI decreased and BP, urine output, and oxygen requirement improved significantly following HC. Despite the HC group being sicker at baseline, those who received HC had similar survival from septic episodes. However, after adjustment for baseline sickness, a multivariate analysis revealed that receipt of HC and GA were associated with a higher risk of death and/or BPD at 36 weeks PMA - After adjustment for baseline sickness, the HC group had a higher risk of mortality at 1 year PMA (HR: 6.08, *p* = 0.01)
Mizobuchi, 2011, Japan, matched case–control	12	NA	I: 24.6 ± 1.3 C: 24.8 ± 1.3	I: 554 ± 100 C: 669 ± 116	Early (HC initiated along with the first inotrope)	A single HC dose of 2 mg/kg was used. If hypotension persisted, the dose was repeated q 12 h. The duration of HC treatment was not mentioned	- ELGANs within the first 7 days of life were enrolled - Definition of hypotension: MAP <30 mm Hg for infants with GA between 25 and 27 weeks, and <25 mm Hg for infants with GA between 23 and 24 weeks - IBP was used - Volume expansion with 10 ml/kg was given before inotrope initiation. The type of fluid used was not mentioned - The initial and maximum doses of dopamine were not mentioned. HC was given if hypotension persisted despite a dopamine dose of 5 μg/kg/min - Infants <25 weeks GA were given prophylactic indomethacin (0.1 mg/kg of continuous infusion for 12 h) for prevention of both PDA and IVH and were also treated with prophylactic glucose-insulin therapy for prevention of hyperkalemia on the 1st day of life - The MAP in the HC group increased significantly at 2 h after HC treatment and was comparable to the control group at 5 h and remained at normal levels until 12 h after HC treatment
Noori, 2006, USA, prospective (pre-post)	15 preterm infants and 5 term infants	NA	Preterm: 26 (23–34) Term: 39 (37–40)	Preterm: 695 (495–2,095) Term: 3,100 (2,618–3,980)	Late (HC was started at ≥ 15 μg/kg/min of dopamine and/or dobutamine)	HC dose was 2 mg/kg followed by 1 mg/kg q 12 h up to four additional doses. A full course of HC was given only when the patient remained hypotensive or maintained only minimum acceptable BP with a dopamine dose of 5–8 μg/kg/min	- Both term and preterm infants of less than 72 h of age were included - Definition of hypotension: BP values <10th percentile - Both IBP and NIBP were used. BP recordings were reported at different time points after HC initiation from 1 to 48 h - Hypotensive infants received one dose of 10–20 ml/kg of normal saline prior to inotrope initiation - Dopamine dosage used was 5–20 μg/kg/min - Echocardiography was used to monitor the effect of HC on cardiac function - In both the preterm and term neonates, HC improved BP without compromising cardiac function, systemic perfusion, or cerebral and renal blood flow - Hyperglycemia (defined as two consecutive blood sugar values of >200 mg/dl) was significantly higher in the HC group. The requirement for insulin for the treatment of hyperglycemia was comparable between the two groups - The authors also evaluated the outcomes of low vs. high-dose HC therapy with the cut-off being 4 mg/kg/day. There were no significant differences in the outcomes except for cortisol levels and the proportion of infants with hypertension (BP> 90th centile) which was higher in the high-dose group - Infants treated with HC who had pre-treatment cortisol levels >15 μg/dl had significantly higher rates of hyperglycemia, insulin therapy. and death than those with levels <15 μg/dl - The authors concluded that the lowest effective HC dose should be used and that HC therapy should be avoided in infants who are not cortisol deficient
Peeples, 2017, USA, retrospective cohort	70	36	I: 25 ± 1 C: 25 ± 1	I: 687 ± 161 C: 768 ± 174	Late (HC was initiated in neonates requiring a mean vasopressor dose of 12.4 μg/kg/min)	HC dose was 1 mg/kg followed by 0.5–1 mg/kg q 8–12 h. There was variation in the cumulative dosage of HC due to clinician discretion from 1 to 4 mg/kg/d. Duration of HC therapy not mentioned	- Preterm infants <28 weeks who were of postnatal age up to 28 days were enrolled. Except for birth weight, all other major baseline characteristics were similar between the two groups - Definition of hypotension: There was no specific BP cut-off for defining hypotension. It was based on the clinician's discretion based on abnormal perfusion suggested by decreased urine output, acidosis, or clinical evidence of organ dysfunction - No information regarding how BP was monitored - No information as to whether any saline boluses were given preceding inotrope initiation - The HC group had a higher incidence of hypertension (*p* = 0.02) and hyperglycemia (*p* = 0.02). When the high dose (4 mg/kg/day) and. the low dose (1–3 mg/kg/day) were compared, the higher dose group had a significantly higher incidence of hypertension (*p* = 0.02). Further, those with pre-treatment cortisol levels of >15 μg/dl had higher rates of hyperglycemia (*p* = 0.01), insulin therapy (*p* = 0.03), and death (*p* = 0.001) when compared to those with ≤15 μg/dl - The increased incidence of death in the high cortisol group was independent of the HC dose used - Vasopressor requirement decreased significantly in the cortisol-deficient group who were treated with HC over a period of 14 h
Seri, 2001, USA retrospective (pre-post)	21	NA	26.9 ± 3.9	952 ± 607	Late [HC was initiated at 20 μg/kg/min of dopamine or when dopamine (10–15 μg/kg/min) with dobutamine and/or epinephrine failed]	HC dose and duration varied based on the etiology of hypotension. HC dose used was 1 mg/kg/dose q 12 h. Preterm infants with severe capillary leak syndrome and/or previous steroid treatment received 3–6 mg/kg/d divided twice daily or four times daily for 2–3 days for a total of six treatment courses	- Preterm infants of ≤14 weeks postnatal age were included - Definition of hypotension not mentioned - IBP was used. BP recordings were monitored until 24 h - Hypotensive infants received volume resuscitation (dose not mentioned) - Maximum dosage of dopamine used was 20 μg/kg/min or 10–15 μg/kg/min and dobutamine/epinephrine was concurrently used - Response to treatment was evaluated at 24 h

GA, gestational age; BW, birth weight; I, intervention group; C, control group; SD, standard deviation; CDH, congenital diaphragmatic hernia; HC, hydrocortisone; IVH, intraventricular hemorrhage; NEC, necrotizing enterocolitis; PDA, patent ductus arteriosus; BPD, bronchopulmonary dysplasia; BP, blood pressure; MAP, mean arterial pressure; IBP, invasive BP; NIBP, non-invasive BP; ELBW, extremely low birth weight; ELGANs, extremely low gestational age neonates; VAI, vasoactive-inotropic; HR, hazard ratio; PMA, post-menstrual age; NA, not applicable.

**Table 3:** Narrative review of the included studies.

Author, Year, Country, Study design	Intervention (number of participants)	Comparator (number of participants)	GA (weeks) mean ± SD or median (range)	BW (g) mean ± SD or median (range)	Time of initiation of hydrocortisone	Dose and duration of hydrocortisone	Other comments
Baker, 2008, USA, retrospective study	117	NI	35 (26–39)	2,620 (880–3,400)	Late (corticosteroids initiated when highest dosages of dopamine and dobutamine were reached)	Stress dose 45 mg/m^2^/d, divided every 6 h for the first 48 h regardless of the baseline cortisol concentration. The maintenance dose of HC was 15 mg/m^2^/day divided every 6 h based on the baseline serum cortisol concentration	- All infants with refractory hypotension were included - The maintenance dose of HC was given only when the baseline cortisol level (12 h after stress dose) was <5 μg/dl and/or hemodynamically unstable - Definition of hypotension: MAP < GA - IBP and/or NIBP were used - Treatment with HC increased the MAP at 2, 6, 12, and 24 h after initiation; decreased the total inotrope dose at 6, 12, and 24 h; and was associated with the resolution of oliguria - No difference was observed for the outcomes of IVH > grade 2, PVL, bacterial or fungal sepsis, and spontaneous intestinal perforation after HC treatment
Batton, 2012, USA, RCT	Pilot study enrolling 10 infants (2×2 factorial study)	NA	23^0/7^–26^6/7^	NA	Early [enrolled infants were administered study infusion (dopamine or placebo) and study syringe medication (HC or placebo)]	Initial dose of 1 mg/kg followed by six doses of 0.5 mg/kg q 12 h	- Only preterm infants <27 weeks within the first 24 h were included - Definition of hypotension: protocol-driven low BP from various centers - Studied infants received two doses of crystalloid at ≤20 ml/kg before randomization - Enrolled infants were administered study infusion (dopamine or placebo) and study syringe medication (hydrocortisone or placebo) - 2 × 2 factorial randomization: (1) dopamine/placebo; (2) dopamine/hydrocortisone; (3) placebo/placebo; (4) placebo/hydrocortisone - 6 μg/kg/min of dopamine (or placebo volume equivalent) was initiated and increased every 20 min to a maximum rate of 15 μg/kg/min - Only 17% of eligible infants were enrolled and enrollment of the study subjects was poor - None of the infants received open-label anti-hypotensive therapy in the first postnatal week - Two infant deaths occurred in the placebo/placebo group, the time of death was distant from enrollment (days 20 and 62)
Bourchier, 1997, New Zealand, RCT	21	19	I: 26.6 ± 2.1 C: 27.5 ± 1.6	I: 923 ± 188 C: 1,043 ± 184	Early (HC was started before inotrope initiation)	An initial dose of HC (2.5 mg/kg q 4 h) was continued for 48 h, followed by 1.25 mg/kg for 48 h, then 0.625 mg/kg for 48 h	- VLBW infants of postnatal age less than 7 days were enrolled - Definition of hypotension: MAP <25 mm Hg (birthweight 500–749 g), <30 mm Hg (BW 750–999 g), 35 mm Hg (BW 1,000–1,499 g) on two occasions 30 min apart - The method of BP measurement (IBP or NIBP) was not mentioned - Infants in both groups received a dose of colloid at 10 ml/kg prior to randomization - In the I group (HC alone), persistent hypotension after the first dose of HC was managed by a dose of colloid. If hypotension continued or developed after the second dose of HC, this was judged as treatment failure and the infant was started on dopamine - In the C group (inotrope alone), dopamine was started at 5 μg/kg/min, increasing stepwise to a maximum dose of 20 μg/kg/min if necessary. If hypotension persisted, noradrenaline infusion (0.05–0.5 μg/kg/min) was added. Once normotension was achieved for 24 h, the inotropes were reduced over a 24–48 h period - There was no pre- or post-correlation baseline cortisol (after ACTH stimulation) or maximum dopamine in either of the two groups - Slower weaning of HC (over 144 h) resulted in hypertension in four neonates - There were four treatment failures in HC group who were successfully treated with dopamine - There was no difference in the outcomes of survival, BPD, ROP (stage 2–4), IVH (grades 2–4), NEC, symptomatic PDA, insulin requirement, and sepsis
Heckman, 1999, Germany, retrospective (pre-post)	30	NA	26.0 ± 1.7	NA	Early (HC started prior to the initiation of the first inotrope)	HC dose used was 2–3 mg/kg/dose. Duration and frequency of dosing were not mentioned	- Only preterm infants <28 w within the first 72 h were included - Definition of hypotension: MAP <25 mm Hg (birthweight 500–749 g), <30 mm Hg (BW 750–999 g), 35 mm Hg (BW 1,000–1,499 g) on two occasions 30 min apart - IBP/NIBP were not mentioned - Studied infants received two doses of crystalloid at 15 ml/kg before HC and/or dopamine initiation - Dopamine was initiated at 5 μg/kg/min and the maximum dose was not mentioned - Of the 30 hypotensive infants, only 16 required dopamine, and four out of the 16 infants were weaned off dopamine within the first 12 h of HC initiation
Helbock, 1993, USA, prospective study (pre-post)	6	NI	NI	510–750	Late (HC after the highest dosage of vasopressor therapy was required)	24–60 mg/m^2^/day was used. Duration was not specified	- Six ELBW neonates were evaluated - Definition of hypotension: MAP < 32 mm Hg - IBP was used - HC was added once the MAP was 21 mm Hg despite the addition of 10–50 ml/kg of volume expansion (saline, 5% albumin, or packed blood red blood cells) and 24 to 60 μg/kg/min of cumulative inotrope therapy - Four of the enrolled neonates had cortisol concentrations <5 μg/dl - Increase in BP occurred as early as 30 min following the administration of HC, and within 12–30 h all the newborns were normotensive without inotrope support
Krediet, 1998, Holland, RCT	13	13	NI	NI	Late (HC was initiated if the neonate was hypotensive despite receiving dopamine and/or dobutamine at 20 μg/kg/min)	5 mg/kg/d in four divided doses	- Only the abstract was available - Definition of hypotension: MAP < GA - No difference in additional requirement of inotropes or volume expanders was found - In the HC group, the increase in MAP to normal values occurred more rapidly (within 6 h) - No differences were found between groups for IVH
Rios, 2014, USA, retrospective cohort	1,501	4,033	I: 32 ± 6.2 C:33 ± 5.4	I: 2,047 ± 1,232 C:2,136 ± 1,106	NI	Dose of HC not mentioned	- Both preterm and term infants within the first 28 days of life were evaluated - Definition of hypotension: International Classification of Disease, Ninth Revision diagnosis code for hypotension (458.0, 458.1, 458.2, 458.8, or 458.9) - NI regarding how BP was monitored - NI as to whether any saline boluses were given preceding inotrope initiation - The trend of use of inotropes between 2001 and 2012 was reported. The most used inotrope was dopamine (65.3%), followed by dobutamine (19.9%), HC (18.8%), epinephrine (16.6%), vasopressin (0.8%), norepinephrine (0.6%), and phenylephrine (0.4%) - Whilst dopamine and dobutamine usage declined, the use of HC and vasopressin increased - For the sub-group of ELBW infants, the most commonly used inotrope was dopamine (83.4%) followed by HC (33%), epinephrine (32.8%), dobutamine (28.4%), phenylephrine (0.9%), vasopressin (0.7%), and norepinephrine (0.3%). The use of dobutamine declined, and the use of HC substantially increased over the study period - There was no significant improvement in survival of hypotensive neonates during the 10-year study period - Those neonates who required ≥4 inotropes had a >60% mortality rate
Robertson, 2017, USA, retrospective study	53	102	I: 38.2 ± 1.8 C: 38.3 ± 1.6	I: 3,021.8 ± 565 C: 3,037.2 ± 576	NI	1–1.5 mg/kg every 6 h for 2 days followed by 0.5 mg/kg every 6 h until the clinical situation necessitates	- Neonates ≥34 weeks of gestation with CDH were included - Definition of hypotension not reported - IBP/NIBP not mentioned - Use of the volume expander was not mentioned. - Dose of inotropes, and the titration and initiation of steroid not mentioned - Patients treated with HC had refractory hypotension (definition not mentioned) and included a significantly sicker group of infants (low APGAR score at 1 and 5 min and type C and D CDH defect) - HC stress therapy was initiated within 48 h of delivery in 58.5% of the patients - Total duration of HC averaged 17.8 ± 14.5 days - Mortality was significantly higher for those treated with HC as expected - No significant differences were found in survival between the high and low cortisol groups, but mortality trended higher in patients with high cortisol levels who received HC - After multivariate analysis, the HC stress dose administration duration was associated with an increased risk of mortality, and the total duration of HC treatment was associated with an increased risk of sepsis
Ramanathan, 1996, USA, retrospective study	19	15	I: 25 ± 2 C: 25 ± 2	I: 705 ± 172 C: 659 ± 124	Late (corticosteroids initiated when highest dosages of dopamine and dobutamine were reached)	NI	- ELBW infants were included - Only the abstract was available - I: HC with inotropes, C: Dexamethasone with inotropes - Definition of hypotension: NI - In all infants, inotropes could be weaned off within 12–48 h after corticosteroid initiation - Serum cortisol levels were low and the levels did not correlate with the degree of hypotension - *Candida* infection developed in one infant in the dexamethasone group, compared to nine infants in the HC group - No difference in death and incidence of bacterial sepsis was noted between the groups
Vishveshwara, 1996, USA, prospective (pre-post study)	41	NI	NI	2,297 ± 893	Late (HC was initiated when the requirement of dopamine was >17 μg/kg/min)	10 mg/kg followed by 5 mg/kg q 8 h for 2 days	- Only the abstract was available - HC was significantly efficacious in reducing the dose of dopamine and in maintaining the BP within the target limits
Verma, 2017, USA, retrospective study	69 (refractory hypotension)	74 (non-refractory hypotension)	I: 25.1 ± 1.3 C: 26.1 ± 1.2	I: 676.1 ± 121.4 C: 734.2 ± 141.4	Late (HC was started when the maximum dose of dopamine, dobutamine, and/or epinephrine was required)	2–4 mg/kg/d (Duration not defined)	- ELBW infants with refractory hypotension were included. Refractory hypotension was defined as hypotension persisting despite treatment with maximum doses of dopamine, dobutamine (20 μg/kg/min), and/or epinephrine (0.5–1 μg/kg/min) in addition to volume expansion - Definition of hypotension: MAP < GA - IBP was used - Hypotensive infants received 10–20 ml/kg of normal saline or 5% albumin prior to inotrope initiation (the number of boluses was not defined and was based on the decision of the clinical team) - Infants in the HC group had lower BW, GA, and higher mean airway pressure and oxygen requirements - The receipt of ANS and incidence of maternal GDM were lower in the HC group - The HC group had a higher risk for IVH which declined in the multivariate analysis - Hypotension in ELBW infants who had a GA of ≤25 and were unexposed to ANS and GDM were refractory to inotrope therapy. The authors concluded that such neonates may benefit from an initial therapy with, or earlier institution of hydrocortisone - No difference in BPD, PDA, NEC, and SIP was found between the two groups

GA, gestational age; BW, birth weight; I, intervention group; C, control group; SD, standard deviation; CDH, congenital diaphragmatic hernia; HC, hydrocortisone; ANS, antenatal corticosteroids; GDM, gestational diabetes mellitus; IVH, intraventricular hemorrhage; BPD, bronchopulmonary dysplasia; NEC, necrotizing enterocolitis; PDA, patent ductus arteriosus; SIP, spontaneous intestinal perforation; BP, blood pressure; MAP, mean arterial pressure; IBP, invasive BP; NIBP, non-invasive BP; ELBW, extremely low birth weight; NA, not applicable; NI, no information.

### 3.1 Risk of bias

Except for two RCTs (14, 34), all the others had a low risk of overall bias. Whilst one had some issues regarding the domain of measurement of the outcome (14), the other had some issues regarding selective reporting (34) (Supplementary Table S3a).

Whilst only two non-RCTs had a moderate risk of overall bias (36, 41), all the other observational studies had a serious risk of overall bias. The predominant reasons for the studies being adjudged as having a serious risk of bias were due to confounding and issues with the classification of interventions (Supplementary Table S3b).

### 3.2 Outcomes

#### 3.2.1 Primary outcomes

##### 3.2.1.1 Mortality

**Figure 2:**
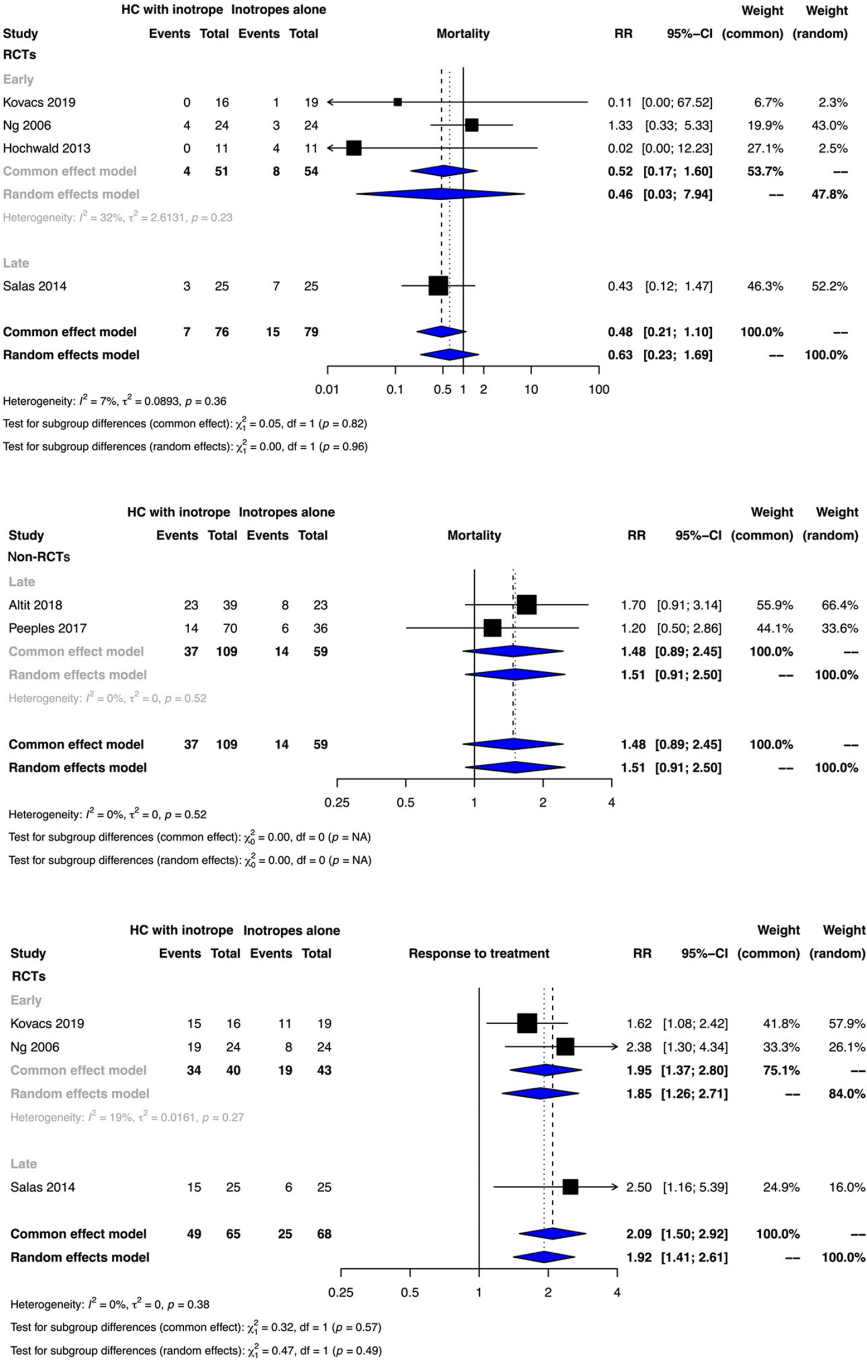
Forest plots depicting the outcomes of mortality and treatment response from randomized controlled trials (RCTs) and non-RCTs.

The meta-analyses of RCTs indicated that clinical benefit or harm could not be ruled out for the outcome of mortality with the use of early or late hydrocortisone along with inotropes when compared to inotropes alone as the effect estimates were statistically non-significant and the certainty of evidence was very low [early initiation risk ratio (RR): 0.46, 95% confidence interval (CI): 0.03–7.92; late initiation RR: 0.43, 95% CI: 0.12–1.47)]. No observational study reported the outcome of mortality for the use of early hydrocortisone (Figure 2, Supplementary Tables S4, S5). The results from non-RCTs were similar to those of RCTs for late hydrocortisone administration (Figure 2, Supplement Table S5).

##### 3.2.1.2 Response to therapy (as defined by the authors)

The very low certainty of evidence suggested that early and late hydrocortisone administration possibly improved the response to inotrope therapy (early initiation RR: 1.85, 95% CI: 1.26–2.71; late initiation RR: 2.50, 95% CI: 1.16–5.39) (Figure 2, Supplementary Tables S4, S5).

#### 3.2.2 Secondary outcomes

##### 3.2.2.1 Clinical outcomes

Clinical benefit or harm could not be ruled out for any of the clinical outcomes from the meta-analyses of RCTs for either early or late hydrocortisone due to statistically non-significant effect estimates and very low to low evidence certainty (Supplementary Figures S1, S2, Tables S4, S5).

The very low certainty of evidence from an observational study indicated that the late hydrocortisone therapy possibly increased the risk of NEC ≥ stage 2 (RR: 2.46, 95% CI: 1.19–5.08) (Supplementary Figure S1, Table S2).

##### 3.2.2.2 Surrogate outcomes

###### 3.2.2.2.1 Change in BP

**Figure 3:**
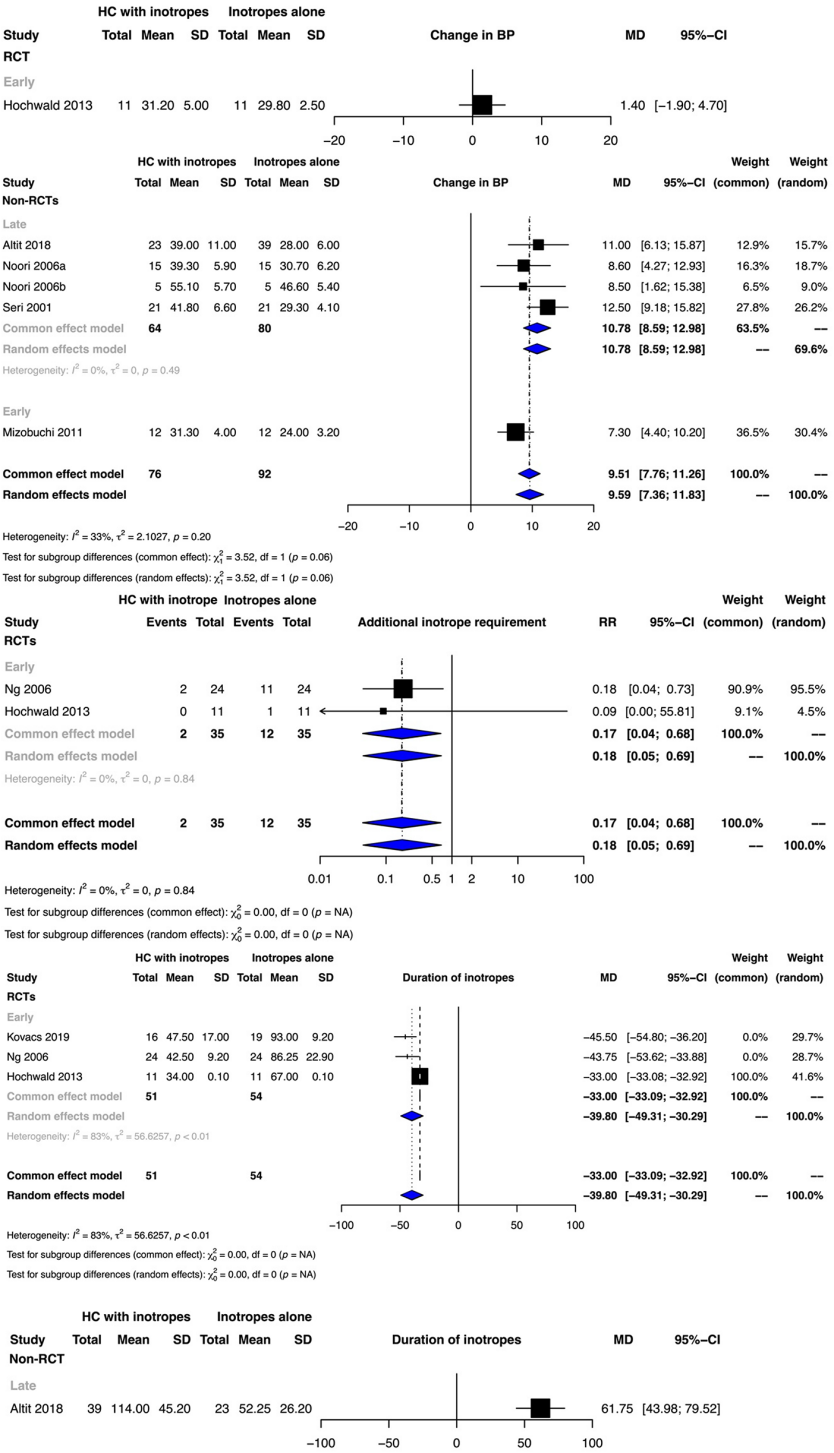
Forest plots depicting the outcomes of additional inotrope requirement, change in blood pressure (BP,) and inotrope duration from randomized controlled trials (RCTs) and non-RCTs.

Clinical benefit or harm could not be ruled out for this outcome for early hydrocortisone therapy as reported from a single RCT (Figure 3). Meta-analyses of non-RCTs indicated that the administration of hydrocortisone in addition to vasopressor therapy either early or late possibly increased the mean BP, with the evidence certainty being very low [early initiation mean difference (MD): 10.78 mm Hg, 95% CI: 4.40–10.20); late initiation MD: 10.78 mm Hg, 95% CI: 8.59–12.98) (Figure 3, Supplementary Tables S4, S5).

###### 3.2.2.2.2 Requirement of additional inotropes

The low certainty of evidence suggested that early hydrocortisone therapy possibly decreased the risk of the requirement of additional inotropes (RR: 0.18, 95% CI: 0.05–0.69) (Figure 3, Supplementary Table S4).

###### 3.2.2.2.3 Duration of inotropes (hours)

Whilst early hydrocortisone initiation possibly decreased the duration of inotrope therapy (MD: −39.8, 95% CI: −30.29 to −49.31; very low certainty), late hydrocortisone initiation possibly increased the duration (MD: 61.75 h, 95% CI: 43.98–79.52; low certainty) (Figure 3, Supplementary Tables S4, S5). While clinical benefit or harm could not be ruled out for the duration of hospitalization for early hydrocortisone therapy from the meta-analysis of two RCTs, one observational study indicated that late hydrocortisone therapy possibly increased the duration of hospitalization (MD: 38.00 days, 95% CI: 12.11–63.89; low certainty) (Supplementary Figure S2, Tables S4, S5).

Clinical benefit or harm could not be ruled out for the outcome of duration of ventilation for early and late hydrocortisone administration (Supplementary Figure S2, Supplementary Tables S4, S5).

###### 3.2.2.2.4 Hyperglycemia

The study by Ng et al. found a higher incidence of glycosuria in the hydrocortisone-treated group (*p* = 0.03) (35).

###### 3.2.2.2.5 Correlation with cortisol levels

Bourchier and Weston reported that there was no correlation between the levels of cortisol (after ACTH stimulation) either at baseline or during therapy and the maximum dopamine dosage (14). However, Kovacs et al. reported that baseline cortisol levels were low (<15 μg/dl) in both the randomized groups (early hydrocortisone initiation with inotropes vs. inotropes alone) and that the levels in the inotropes alone group progressively decreased during treatment (*p* = 0.02) (15). Three observational studies indicated that late hydrocortisone therapy in neonates with high baseline cortisol levels may be associated with an increased risk of mortality and other adverse events such as hyperglycemia requiring insulin therapy, hypertension, and mortality (13, 29, 37).

### 3.3 Narrative review

Amongst the eight studies included in the narrative review, all except one had evaluated late hydrocortisone therapy. (Table 3) Of the three RCTs included in the descriptive review (14, 31, 34), two had evaluated early hydrocortisone therapy (14, 31). Most of the studies reported an increase in mean BP and a reduction in inotrope usage after hydrocortisone initiation. Robertson et al. studied the use of late hydrocortisone therapy in neonates diagnosed with congenital diaphragmatic hernia (CDH) and reported that after adjusting for baseline sickness, late hydrocortisone therapy was associated with an increased risk of mortality, and that the total duration of hydrocortisone treatment was associated with an increased risk of sepsis (13). Ramanathan et al. concluded that the late administration of hydrocortisone was associated with an increased risk of fungal sepsis (38). Verma et al., in their multivariate analysis, found that late administration of hydrocortisone was associated with an increased risk of IVH in preterm neonates of ≤25 weeks of gestation (40). Helbock et al., who studied late hydrocortisone therapy, reported that four of the six hypotensive ELBW neonates had a baseline cortisol level of <5 μg/dl (12). However, Robertson et al. did not find any statistically significant difference in survival between hypotensive infants with CDH and baseline cortisol levels. The characteristics of the studies included in the narrative review are provided in Table 3.

The EtD framework and the recommendations are provided in Supplementary Tables S6, S7.

## 4 Discussion

This systematic review and meta-analysis indicated that early hydrocortisone therapy in neonates who require vasopressor therapy was associated with improved outcomes with no major adverse events when compared to the late administration of hydrocortisone.

The results of this meta-analysis indicated that clinical benefit or harm could not be ruled out for the outcome of mortality for either of the interventions. However, Altit et al., Peeples, and Noori et al. indicated that late hydrocortisone therapy possibly increased the risk of mortality (29, 36, 37). The results from these studies could not be pooled and were considered in the EtD framework when we formulated the recommendations. For the outcome of the response to therapy, both early and late hydrocortisone therapy as an adjunct to inotropes were possibly beneficial. Further, late HC therapy was possibly associated with an increased duration of inotrope therapy. Multiple studies in adult populations have reported that prolonged exposure to inotrope therapy is associated with an increased risk of mortality (43–45). We hypothesize that the prolonged exposure to inotropes in the late hydrocortisone group may have contributed to increased mortality. One non-RCT included in our systematic review indicated that late hydrocortisone therapy was possibly associated with an increased risk of NEC ≥ stage 2. Two observational studies included in this systematic review also suggested that late hydrocortisone administration was possibly associated with an increased risk of sepsis (13, 38). Similarly, Hotta et al. also indicated that exposure to dopamine was associated with an increased risk of sepsis in ELGANs, even after adjusting for baseline sickness, which could explain the findings of our systematic review (46).

The results of this systematic review were contentious with respect to baseline cortisol levels and their correlation with response to hydrocortisone therapy or adverse events. Whilst one RCT that had enrolled ELGANs suggested no correlation (14), the other that had included a relatively mature group of infants indicated that the cortisol levels progressively decreased in the placebo group (15). The discrepancy in the results could be attributed to the difficulty in interpreting cortisol levels in neonates as it is complicated by several factors (2). The results from observational studies predominantly suggested that late hydrocortisone therapy in neonates with high baseline cortisol levels was possibly associated with an increased risk of adverse effects including mortality (13, 29, 37). The patient population enrolled in these studies was widely heterogeneous, including ELGANs, infants with hypoxic-ischemic encephalopathy treated with therapeutic hypothermia, and those who were diagnosed with CDH. None of the included studies had evaluated long-term neurodevelopmental outcomes. However, prophylactic low-dose hydrocortisone therapy in ELGANs at the highest risk of mortality or BPD has been studied extensively (47–50). Some of these studies used hydrocortisone for a prolonged period, even up to 15 days at relatively high cumulative dosages (up to >10 mg/kg) (47, 51). Most of these studies indicated improved long-term neurodevelopmental outcomes with prophylactic hydrocortisone, though the patient population evaluated in these studies differed from ours (52–54). Unlike dexamethasone, which only has glucocorticoid activity, suppresses endogenous cortisol, and, hence downregulates the mineralocorticoid receptor stimulation of neurons through endogenous cortisol, resulting in neuronal apoptosis, hydrocortisone acts similar to endogenous cortisol with the stimulation of both glucocorticoid and mineralocorticoid receptors. This might be one of the possible reasons for better long-term neurodevelopmental outcomes with hydrocortisone when compared to dexamethasone.

After careful consideration of the aspects specified in the EtD framework, the guideline development group suggested that early hydrocortisone may be used in the treatment of neonates with fluid refractory shock as an adjunct to inotropes. We suggest initiating hydrocortisone therapy when there is an increased requirement for inotropes. The most commonly used first-line inotrope included in this systematic review was dopamine, and hydrocortisone therapy may be initiated if the neonate requires a dopamine dosage of ≥10 μg/kg/min, which translates to a VIS of 10. There is insufficient evidence to recommend the timing of the introduction of hydrocortisone in relation to other inotropes such as epinephrine, norepinephrine, vasopressin, and milrinone when used as a first-line treatment. In such scenarios, dopamine equivalent doses adjudged according to the VIS may be considered. The equivalent doses would be dobutamine ≥10 mcg/kg/min, epinephrine ≥0.1 mcg/kg/min, norepinephrine ≥0.1 mcg/kg/min, vasopressin ≥0.0001 IU/kg/min, and milrinone ≥1 μg/kg/min. This is a weak recommendation with very low evidence certainty.

The dosage of hydrocortisone utilized in the studies varied widely. In preterm neonates, 1 mg/kg followed by 0.5–1 mg/kg every 8–12 h of hydrocortisone may be considered. Whereas in term neonates, a dose of 2 mg/kg followed by 1 mg/kg every 6–8 h may be used. Further, hydrocortisone may be tapered over 2–3 days once the desired effect has been achieved, i.e., when the neonate is being weaned from inotropes.

To the best of our knowledge, this is the only clinical practice guideline on the timing of instituting hydrocortisone therapy in neonates with shock. There were several limitations to this systematic review. First, it was a pragmatic review with a widely disparate patient population. Second, the dosage of hydrocortisone utilized was different between the included studies. We addressed the first two limitations by downrating the level of evidence for the indirectness related to the patient population domain and the intervention. Finally, we only included published literature.

## 5 Conclusions

Earlier use of hydrocortisone in neonates with shock is possibly associated with better outcomes with no significant adverse effects. Later administration of hydrocortisone in neonates with shock may be associated with an increased risk of mortality, NEC, and prolonged duration of inotropes and hospital stay. The recommendations were predominantly based on very low evidence certainty which indicates the need for adequately powered multi-center trials that evaluate the timing of introduction of hydrocortisone in neonatal shock.

## Data Availability

The original contributions presented in the study are included in the article/Supplementary Material, further inquiries can be directed to the corresponding author.
