# Supplementary material for: Timing of hydrocortisone therapy in neonates with shock: a systematic review, meta-analysis, and clinical practice guideline
Source: Front Pediatr. 2025 Mar 12;13:1491976. doi: 10.3389/fped.2025.1491976 (PMC11937126; doi:10.3389/fped.2025.1491976)
Supplement: Supplementary file 1 [file Datasheet1.pdf]

Supplement file

Supplement Figure 1: Forest plots depicting the outcomes major brain injury (MBI), necrotising enterocolitis (NEC) ≥ stage 2 and culture proven sepsis from randomized controlled trials (RCTs) and non-RCTs.

Supplement Figure 2: Forest plots depicting the outcomes patent ductus arteriosus (PDA) requiring intervention, retinopathy of prematurity (ROP) requiring intervention, bronchopulmonary dysplasia (BPD), duration of ventilation and hospitalisation from randomized controlled trials (RCTs) and non- RCTs.

Supplement Table 1: Literature search strategy.

Supplement Table 2: Statements to communicate the findings of the systematic review.

Supplement Table 3: Risk of bias assessment of RCTs and non-RCTs.

Supplement Table 4: Summary of Findings table for the GRADE certainty of evidence assessment for early hydrocortisone therapy.

Supplement Table 5: Summary of Findings table for the GRADE certainty of evidence assessment for late hydrocortisone therapy.

Supplement Table 6: Evidence to Decision (EtD) framework for early hydrocortisone therapy.

Supplement Table 7: Evidence to Decision (EtD) framework for late hydrocortisone therapy.

Supplement Figure 1: Forest plots depicting the outcomes major brain injury (MBI), necrotising enterocolitis (NEC) ≥ stage 2 and culture proven sepsis from randomized controlled trials (RCTs) and non-RCTs

Study	HC with inotrope Events	HC with inotrope Total	Inotropes alone Events	Inotropes alone Total	RR	95%-CI	Weight (common)	Weight (random)
**RCTs**								
Early								
Ng 2006	4	24	6	24	0.67	[0.22; 2.07]	68.6%	62.3%
Kovacs 2019	3	16	3	19	1.19	[0.28; 5.09]	31.4%	37.7%
Common effect model	**7**	**40**	**9**	**43**	**0.83**	**[0.34; 2.01]**	**100.0%**	**--**
Random effects model					**0.83**	**[0.34; 2.02]**	**--**	**100.0%**
Heterogeneity: I^2 = 0%, τ^2 = 0, p = 0.54								
**Common effect model**	**7**	**40**	**9**	**43**	**0.83**	**[0.34; 2.01]**	**100.0%**	**--**
**Random effects model**					**0.83**	**[0.34; 2.02]**	**--**	**100.0%**

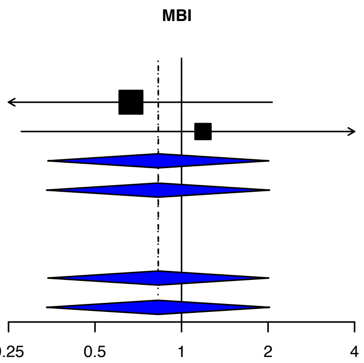

Heterogeneity: I^2 = 0%, τ^2 = 0, p = 0.54
Test for subgroup differences (common effect): χ^2_0 = 0.00, df = 0 (p = NA)
Test for subgroup differences (random effects): χ^2_0 = 0.00, df = 0 (p = NA)

Study	HC with inotrope Events	HC with inotrope Total	Inotropes alone Events	Inotropes alone Total	RR	95%-CI	Weight (common)	Weight (random)
**RCTs**								
Early								
Kovacs 2019	0	16	0	19			0.0%	0.0%
Ng 2006	2	24	3	24	0.67	[0.12; 3.64]	50.0%	60.6%
Hochwald 2013	1	11	3	11	0.33	[0.04; 2.73]	50.0%	39.4%
Common effect model	**3**	**51**	**6**	**54**	**0.50**	**[0.14; 1.84]**	**100.0%**	**--**
Random effects model					**0.51**	**[0.14; 1.90]**	**--**	**100.0%**
Heterogeneity: I^2 = 0%, τ^2 = 0, p = 0.61								
**Common effect model**	**3**	**51**	**6**	**54**	**0.50**	**[0.14; 1.84]**	**100.0%**	**--**
**Random effects model**					**0.51**	**[0.14; 1.90]**	**--**	**100.0%**

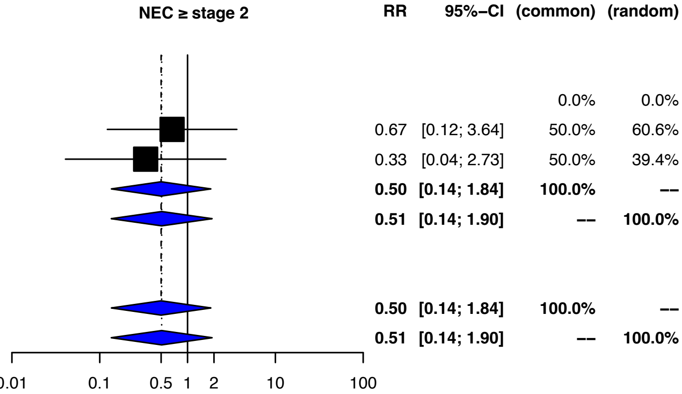

Heterogeneity: I^2 = 0%, τ^2 = 0, p = 0.61
Test for subgroup differences (common effect): χ^2_0 = 0.00, df = 0 (p = NA)
Test for subgroup differences (random effects): χ^2_0 = 0.00, df = 0 (p = NA)

Study	HC with inotrope Events	HC with inotrope Total	Inotropes alone Events	Inotropes alone Total	RR	95%-CI
**Non-RCT**						
Late						
Altit 2018	25	39	6	23	2.46	[1.19; 5.08]

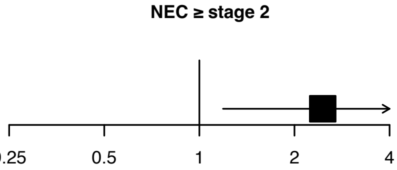

Study	HC with inotrope Events	HC with inotrope Total	Inotropes alone Events	Inotropes alone Total	RR	95%-CI	Weight (common)	Weight (random)
**RCTs**								
Early								
Hochwald 2013	3	11	5	11	0.60	[0.19; 1.92]	61.6%	79.8%
Kovacs 2019	1	16	1	19	1.19	[0.08; 17.51]	11.3%	14.9%
Ng 2006	1	24	0	24	11.00	[0.02; 6951.92]	1.2%	2.6%
Common effect model	**5**	**51**	**6**	**54**	**0.86**	**[0.31; 2.37]**	**74.1%**	**--**
Random effects model					**0.72**	**[0.25; 2.06]**	**--**	**97.3%**
Heterogeneity: I^2 = 0%, τ^2 = 0, p = 0.60								
Late								
Salas 2014	0	25	2	25	0.05	[0.00; 26.45]	25.9%	2.7%
**Common effect model**	**5**	**76**	**8**	**79**	**0.65**	**[0.25; 1.72]**	**100.0%**	**--**
**Random effects model**					**0.67**	**[0.24; 1.89]**	**--**	**100.0%**

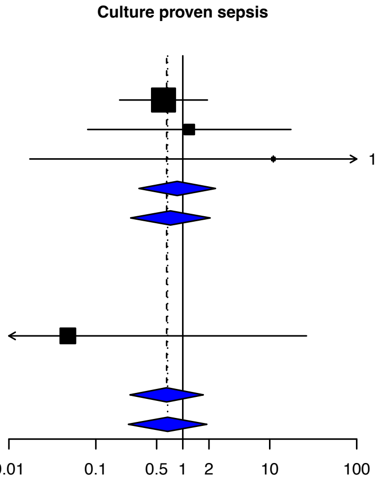

Heterogeneity: I^2 = 0%, τ^2 = 0, p = 0.66
Test for subgroup differences (common effect): χ^2_1 = 0.79, df = 1 (p = 0.38)
Test for subgroup differences (random effects): χ^2_1 = 0.69, df = 1 (p = 0.41)

Study	HC with inotrope Events	HC with inotrope Total	Inotropes alone Events	Inotropes alone Total	RR	95%-CI
**Non-RCT**						
Late						
Altit 2018	35	39	21	23	0.98	[0.83; 1.16]

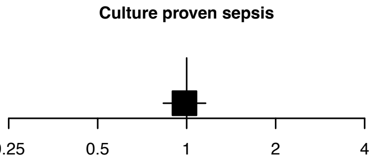

Supplement Figure 2: Forest plots depicting the outcomes patent ductus arteriosus (PDA) requiring intervention, retinopathy of prematurity (ROP) requiring intervention, bronchopulmonary dysplasia (BPD), duration of ventilation and hospitalisation from randomized controlled trials (RCTs) and non-RCTs

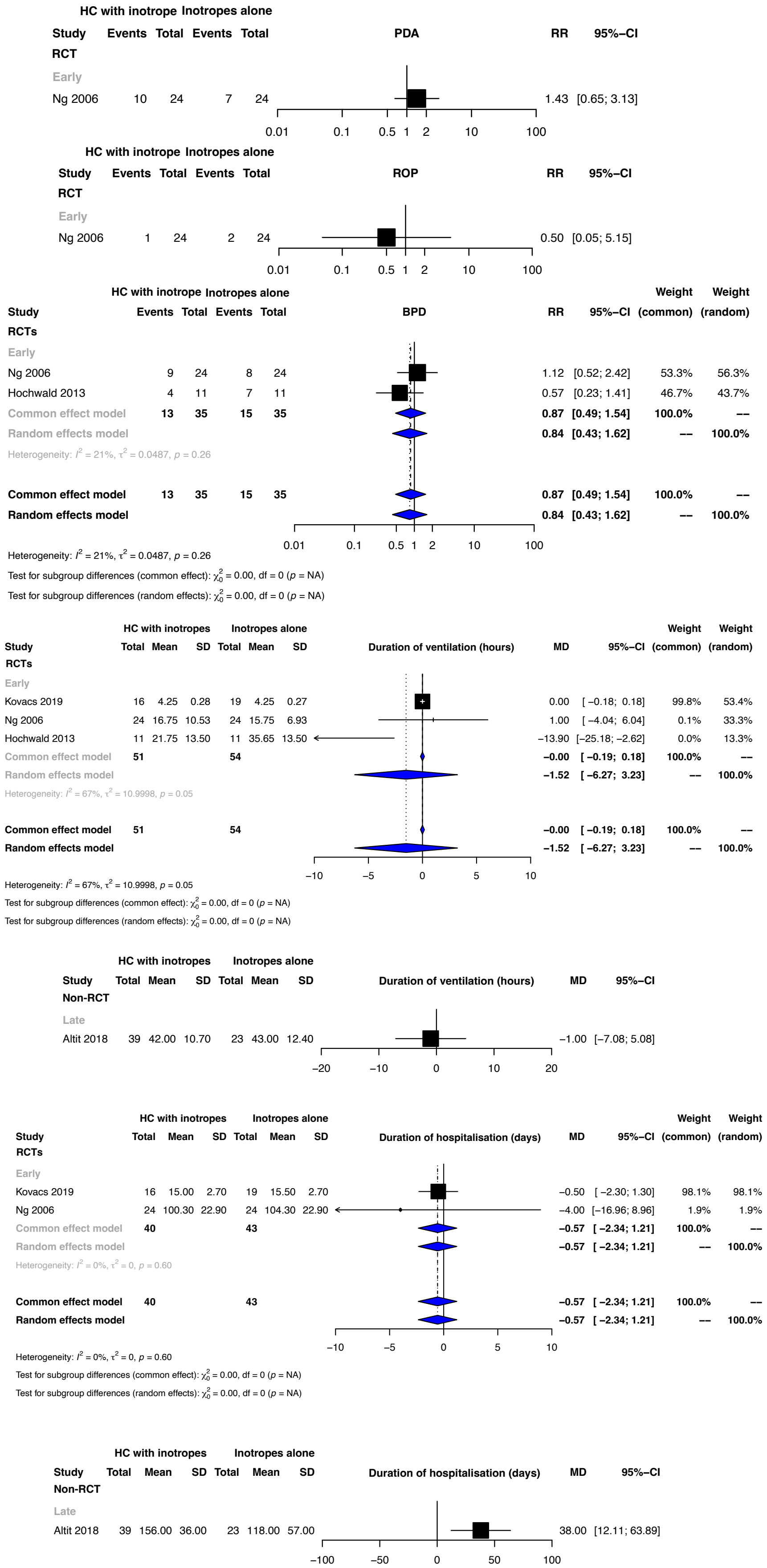

Supplement Table 1: Literature search strategy.

#	Searches	Medline Results	Embase Results
**1**	newborn.mp. or Infant, Newborn/	821660	827831
**2**	(Neonat* or Newborn* or Preterm* or term or premature or "Low birth weight" or lbw or vlbw or elbw or "Low birth weights" or "Low birthweight" or "Low birthweights" or Infant* or Infant* or "Small gestational age" or SGA or "Extremely premature").ab,ti.	2159448	3023435
**3**	1 or 2	2509981	3308084
**4**	"shock*".ab,ti.	217857	314438
**5**	exp Shock, Cardiogenic/ or exp Shock/	85657	9778
**6**	(circulatory adj3 (failur* or collapse or insufficienc*)).ab,ti.	5748	180621
**7**	4 or 5 or 6	258791	1851510
**8**	3 and 7	20261	34372
**9**	Hydrocortisone.mp. or exp Hydrocortisone/	84109	177825
**10**	Adrenal Cortex Hormones.mp. or exp Adrenal Cortex Hormones/	423900	1210223
**11**	(adrenal cortex hormone or adrenal cortex hormones or cortex hormone, adrenal or corticoid or corticoids or corticosteroid or corticosteroids or adrenal cortex hormone*).ab,kf,ti.	135760	214621
**12**	steroid.mp. or exp Steroids/	1015334	2027414
**13**	exp Dexamethasone/ or dexamethasone.mp.	80482	207890
**14**	9 or 10 or 11 or 12 or 13	1170162	2067145
**15**	8 and 14	977	3023

Supplement Table 2: Statements to communicate the findings of the systematic review.

Descriptor	Criteria
Clinical benefit/harm	Statistically significant result<br>High certainty evidence<br>Biological mechanism(s) well established<br>Point estimates of underlying studies are consistently in one direction<br>Optimal information size reached[6]
Probable clinical benefit/harm	Statistically significant result<br>Moderate or high certainty evidence<br>Evidence of biological plausibility<br>Point estimates of underlying studies are predominatly in one direction<br>Close to optimal information size or summary confidence interval is sufficiently narrow to give confidence that the true effect would be clinically meaningful if it is only in the ballpark of the summary estimate.
Possible clinical benefit/harm	Statistically significant result<br>Low or very low certainty evidence<br>Few studies, wide summary confidence interval or effect is driven by one or two heavily weighted studies
Improbable benefit/harm	Statistically non-significant result<br>Moderate or high certainty evidence<br>Point estimates of underlying studies are close to and on both sides of the line of null effect
No clinical benefit/harm	Statistically non-significant result<br>High certainty evidence<br>Point estimates of underlying studies are close to and on either side of the line of null effect<br>Majority of underlying studies are adequately powered for outcome of interest<br>Optimal information size reached reached
Clinical benefit/harm cannot be excluded	Statistically non-significant result<br>Low or very low certainty evidence<br>Few studies<br>Wide confidence intervals
Modified GRADE recommendations (Ref: Santesso N, Glenton C, Dahm P, Garner P, Akl EA, Alper B, Brignardello-Petersen R, Carrasco-Labra A, De Beer H, Hultcrantz M, Kuijpers T, Meerpohl J, Morgan R, et al., GRADE Working Group. GRADE guidelines 26: informative statements to communicate the findings of systematic reviews of interventions. J Clin Epidemiol. 2020 Mar;119:126-135.)	

Supplement Table 3a: Risk of bias assessment of RCTs

Study	Randomization process	Deviations from intended interventions	Missing outcome data	Measurement of the outcome	Selection of the reported result	Overall bias
Batton 2012	Low	Low	Low	Low	Low	Low
Bourchier 1997	Low	Low	Low	Some concerns	Low	Some concerns
Hochwald 2013	Low	Low	Low	Low	Low	Low
Kovacs 2019	Low	Low	Low	Low	Low	Low
Krediet 1998	NI	NI	Low	NI	Some concerns	Some concerns
Ng 2006	Low	Low	Low	Low	Low	Low
Salas 2014	Low	Low	Low	Low	Low	Low

Supplement Table 3b: Risk of bias assessment of non-RCTs

Study	Confounding	Selection	Classification of interventions	Deviation from intended intervention	Missing data	Measurement of outcomes	Selective reporting	Overall
Altit 2018	Serious	Low	Serious	NI	Low	Low	Moderate	Serious
Baker 2008	Serious	Low	Serious	NI	Low	Low	Serious	Serious
Heckman 2001	Low	Low	Serious	NI	Low	NI	NI	Serious
Helbock 1993	NI	Serious	Serious	Low	Low	Low	Serious	Serious
Mizobuchi 2011	Serious	Low	Serious	NI	Low	Low	Moderate	Serious
Noori 2006	Low	Low	Moderate	Low	Low	Low	Moderate	Moderate
Ng 2003	Serious	Low	Moderate	Low	Low	Low	Moderate	Serious
Rios 2014	Serious	Low	Serious	Low	Low	Low	Moderate	Serious
Peeples 2017	Serious	Low	Serious	Low	Low	Low	Moderate	Serious
Ramanathan 1996	Moderate	Low	Serious	Low	Low	Low	Serious	Serious
Robertson 2017	Serious	Low	Serious	Low	Low	Low	Moderate	Serious
Seri 2001	Low	Low	Serious	Low	Low	Low	Serious	Serious
Visveshara 1996	Low	Low	Moderate	Low	Low	Low	NI	Moderate

Supplement Table 4: Early hydrocortisone along with inotrope compared to inotrope alone in neonates with shock.

Certainty assessment							Summary of findings				
Participants (studies) Follow-up	Risk of bias	Inconsistency	Indirectness	Imprecision	Publication bias	Overall certainty of evidence	Study event rates (%)		Relative effect (95% CI)	Anticipated absolute effects	
							With inotrope alone	With early hydrocortisone along with the first inotrope		Risk with inotrope alone	Risk difference with early hydrocortisone along with the first inotrope
**Mortality until discharge (RCTs)**											
105 (3 RCTs)[1,2,3]	not serious	not serious	serious[a]	very serious[b]	none	⊕◯◯◯ Very low	8/54 (14.8%)	4/51 (7.8%)	**RR 0.46** (0.03 to 7.94)	148 per 1,000	**80 fewer per 1,000** (from 144 fewer to 1,000 more)
**Major brain injury (IVH grade > 2 and / or cystic PVL) (RCTs)**											
83 (2 RCTs)[1,3]	not serious	not serious	serious[a]	very serious[b]	none	⊕◯◯◯ Very low	9/43 (20.9%)	9/40 (22.5%)	**RR 0.83** (0.34 to 2.02)	209 per 1,000	**36 fewer per 1,000** (from 138 fewer to 213 more)
**Necrotising enterocolitis ≥ stage 2 (RCTs)**											
105 (3 RCTs)[1,2,3]	not serious	not serious	serious[a]	very serious[b]	none	⊕◯◯◯ Very low	6/54 (11.1%)	3/51 (5.9%)	**RR 0.51** (0.14 to 1.90)	111 per 1,000	**54 fewer per 1,000** (from 96 fewer to 100 more)
**Culture positive sepsis (RCTs)**											
105 (3 RCTs)[1,2,3]	not serious	not serious	serious[a]	very serious[b]	none	⊕◯◯◯ Very low	6/54 (11.1%)	5/51 (9.8%)	**RR 0.86** (0.31 to 2.37)	111 per 1,000	**16 fewer per 1,000** (from 77 fewer to 152 more)
**Retinopathy of prematurity requiring treatment (RCT)**											

48 (1 RCT)[3]	not serious	not serious	not serious	very serious[b]	none	⊕⊕◯◯ Low	1/24 (4.2%)	2/24 (8.3%)	**RR 0.50** (0.05 to 5.15)	42 per 1,000	**21 fewer per 1,000** (from 40 fewer to 173 more)

Patent ductus arteriosus requiring intervention (RCT)

48 (1 RCT)[3]	not serious	not serious	not serious	very serious[b]	none	⊕⊕◯◯ Low	10/24 (41.7%)	7/24 (29.2%)	**RR 1.43** (0.65 to 3.13)	417 per 1,000	**179 more per 1,000** (from 146 fewer to 888 more)

Bronchopulmonary dysplasia (O_2 requirement at 36 weeks' postmenstrual age) (RCTs)

70 (2 RCTs)[2,3]	not serious	not serious	serious[a]	serious[b]	none	⊕⊕◯◯ Low	13/35 (37.1%)	15/35 (42.9%)	**RR 0.84** (0.43 to 1.62)	371 per 1,000	**59 fewer per 1,000** (from 212 fewer to 230 more)

Duration of invasive mechanical ventilation (days) (RCTs)

105 (3 RCTs)[1,2,3]	not serious	serious[c]	serious[a]	serious[b]	none	⊕◯◯◯ Very low	54	51	-		MD **1.52 days lower** (6.27 lower to 3.23 higher)

Duration of hospitalisation (days) (RCTs)

83 (2 RCTs)[1,3]	not serious	not serious	very serious[a]	serious[b]	none	⊕◯◯◯ Very low	43	40	-		MD **0.57 days lower** (2.34 lower to 1.21 higher)

Response to treatment (RCTs)

83 (2 RCTs)[1,3]	not serious	not serious	serious[a]	serious[d]	none	⊕⊕◯◯ Low	19/43 (44.2%)	34/40 (85.0%)	**RR 1.85** (1.26 to 2.71)	442 per 1,000	**376 more per 1,000** (from 115 more to 756 more)

Change in blood pressure (RCT)

22 (1 RCT)[2]	not serious	not serious	not serious	extremely serious[b]	none	⊕◯◯◯ Very low	11	11	-		MD **1.4 mm Hg higher** (1.9 lower to 4.7 higher)
**Change in blood pressure (non-RCT)**											
24 (1 observational study)[4]	very serious[e]	not serious	serious[a]	serious[d]	none	⊕◯◯◯ Very low	12	12	-		MD **7.3 mm Hg higher** (4.4 higher to 10.2 higher)
**Additional inotrope requirement (RCTs)**											
70 (2 RCTs)[2,3]	not serious	not serious	serious[a]	serious[d]	none	⊕⊕◯◯ Low	12/35 (34.3%)	2/35 (5.7%)	**RR 0.18** (0.05 to 0.69)	343 per 1,000	**281 fewer per 1,000** (from 326 fewer to 106 fewer)
**Duration of inotropes (hours) (RCTs)**											
105 (3 RCTs)[1,2,3]	not serious	not serious	serious[a]	serious[d]	none	⊕⊕◯◯ Low	54	51	-		MD **39.8 hours lower** (49.31 lower to 30.29 lower)

CI: confidence interval; **MD:** mean difference; **RR:** risk ratio; **RCT:** Randomized controlled trial; **IVH:** Intraventricular hemorrhage; **PVL:** Periventricular leukomalacia; **O_2:** Oxygen.

Explanations

a. Indirectness related to patient population (term and preterm, baseline sickness) and outcomes (definition of hypotension and response to treatment) between the studies.
b. Optimal information criterion (OIS) not met, and 95% CI crosses the line of no effect.
c. $I^2 > 50\%$.
d. OIS criterion not met.
e. All studies had serious risk of bias.

References

1. Kovacs K, Szakmar E, Meder U, Szakacs L, Cseko A, Vatai B et al. A Randomized Controlled Study of Low-Dose Hydrocortisone Versus Placebo in Dopamine-Treated Hypotensive Neonates Undergoing Hypothermia Treatment for Hypoxic-Ischemic Encephalopathy. J Pediatr. 2019;211:13-19.e3.
2. Hochwald O, Palegra G, Osiovich H. Adding hydrocortisone as 1st line of inotropic treatment for hypotension in very low birth weight infants. Indian J Pediatr. 2014 ;81(8):808-10.
3. Ng PC, Lee CH, Bnur FL, Chan IH, Lee AW, Wong E, et al. A double-blind, randomized, controlled study of a “stress dose” of hydrocortisone for rescue treatment of refractory hypotension in preterm infants. Pediatrics. 2006;117(2):367-75.
4. Mizobuchi M, Yoshimoto S, Nakao H. Time-course effect of a single dose of hydrocortisone for refractory hypotension in preterm infants. Pediatr Int. 2011 ;53(6):881-6.

Supplement Table 5: Late hydrocortisone along with inotropes compared to inotropes alone in neonates with shock.

Certainty assessment							Summary of findings				
Participants (studies) Follow-up	Risk of bias	Inconsistency	Indirectness	Imprecision	Publication bias	Overall certainty of evidence	Study event rates (%)		Relative effect (95% CI)	Anticipated absolute effects	
							With inotropes alone	With late hydrocortisone along with inotropes		Risk with inotropes alone	Risk difference with late hydrocortisone along with inotropes
**Mortality until discharge (RCT)**											
50 (1 RCT)[1]	not serious	not serious	not serious	extremely serious[a]	none	⊕◯◯◯ Very low	7/25 (28.0%)	3/25 (12.0%)	**RR 0.43** (0.12 to 1.47)	280 per 1,000	**160 fewer per 1,000** (from 246 fewer to 132 more)
**Mortality until discharge (non-RCTs)**											
168 (2 observational studies)[2,3]	very serious[b]	not serious	not serious	serious[a]	none	⊕◯◯◯ Very low	14/59 (23.7%)	37/109 (33.9%)	**RR 1.51** (0.91 to 2.50)	237 per 1,000	**121 more per 1,000** (from 21 fewer to 356 more)
**Culture positive sepsis (RCT)**											
50 (1 RCT)[1]	not serious	not serious	not serious	extremely serious[a]	none	⊕◯◯◯ Very low	2/25 (8.0%)	0/25 (0.0%)	not estimable	80 per 1,000	
**Culture positive sepsis (non-RCT)**											
62 (1 observational study)[3]	very serious[b]	not serious	not serious	serious[a]	none	⊕◯◯◯ Very low	21/23 (91.3%)	35/39 (89.7%)	**RR 0.98** (0.83 to 1.16)	913 per 1,000	**18 fewer per 1,000** (from 155 fewer to 146 more)
**Necrotising enterocolitis ≥ stage 2 (non-RCT)**											

62 (1 observational study)[3]	very serious[b]	not serious	not serious	serious[c]	none	⊕◯◯◯ Very low	6/23 (26.1%)	25/39 (64.1%)	**RR 2.46** (1.19 to 5.08)	261 per 1,000	**381 more per 1,000** (from 50 more to 1,000 more)
**Response to treatment (RCT)**											
50 (1 RCT)[1]	not serious	not serious	not serious	serious[c]	none	⊕⊕⊕◯ Moderate	6/25 (24.0%)	15/25 (60.0%)	**RR 2.50** (1.16 to 5.39)	240 per 1,000	**360 more per 1,000** (from 38 more to 1,000 more)
**Change in blood pressure (non-RCTs)**											
144 (3 observational studies)[3,4,5]	serious[d]	not serious	serious[e]	serious[c]	none	⊕◯◯◯ Very low	80	64	-		MD **10.78 mm Hg higher** (8.59 higher to 12.98 higher)
**Duration of inotropes (hours) (non-RCT)**											
62 (1 observational study)[3]	very serious[b]	not serious	not serious	serious[c]	strong association	⊕⊕◯◯ Low	23	39	-		MD **61.75 hours higher** (43.98 higher to 79.52 higher)
**Duration of ventilation (hours) (non-RCTs)**											
62 (1 observational study)[3]	very serious[b]	not serious	not serious	serious[a]	none	⊕◯◯◯ Very low	23	39	-		MD **1 hours lower** (7.08 lower to 5.08 higher)
**Duration of hospitalisation (days) (non-RCT)**											
62 (1 observational study)[3]	very serious[b]	not serious	not serious	serious[c]	strong association	⊕⊕◯◯ Low	23	39	-		MD **38 days higher** (12.11 higher to 63.89 higher)
**CI:** confidence interval; **MD:** mean difference; **RR:** risk ratio; **RCT:** Randomized controlled trial.											

Explanations

a. Optimal information size (OIS) criterion not satisfied and 95% CI crossing the line of no effect.
b. All the studies had a serious risk of overall bias.
c. OIS criterion not met.
d. Of the three studies, two had a serious risk of bias and the other had a moderate risk of bias.
e. There was indirectness related to patient population (term and preterm, baseline sickness) and outcomes (definition of hypotension and response to treatment) between the studies.

References

1. Salas G, Travaglianti M, Leone A, Couceiro C, Rodríguez S, Fariña D. Hydrocortisone for the treatment of refractory hypotension: a randomized controlled trial. An Pediatr (Barc). 2014;80(6):387-93.
2. Peeples ES. An evaluation of hydrocortisone dosing for neonatal refractory hypotension. J Perinatol. 2017 ;37(8):943-946.
3. Altit G, Vigny-Pau M, Barrington K, Dorval VG, Lapointe A. Corticosteroid Therapy in Neonatal Septic Shock-Do We Prevent Death? Am J Perinatol. 2018 ;35(2):146-151.
4. Seri I. Hydrocortisone and vasopressor-resistant shock in preterm neonates. Pediatrics. 2006;117(2):516-8.
5. Noori S, Friedlich P, Wong P, Ebrahimi M, Siassi B, Seri I. Hemodynamic changes after low-dosage hydrocortisone administration in vasopressor-treated preterm and term neonates. Pediatrics. 2006 ;118(4):1456-66.

Supplement Table 6: EtD framework for early hydrocortisone

**Should early hydrocortisone (along with the initiation of the first inotrope or with increasing requirement of the first inotrope) vs. inotrope alone be used in neonates with fluid refractory shock?**	
**POPULATION:**	Neonates with fluid refractory (as defined by the authors) shock.
**INTERVENTION:**	Early hydrocortisone along with inotrope (Hydrocortisone introduced early along with the addition of first inotrope or with increasing requirement of the first inotrope (a cut-off of vasoactive inotropic score of 10 was taken).
**COMPARISON:**	Inotrope alone.
**MAIN OUTCOMES:**	Mortality until discharge (RCTs); Major brain injury (IVH grade > 2 and / or cystic PVL) (RCTs); NEC ≥ stage 2 (RCTs); Culture positive sepsis (RCTs); ROP requiring treatment (RCT); PDA requiring intervention (RCT); BPD (O_2 requirement at 36 weeks' PMA) (RCTs); Duration of invasive mechanical ventilation (days) (RCTs); Duration of hospitalisation (days) (RCTs); Response to treatment (as defined by authors) (RCTs); Change in BP (RCT); Change in BP (non-RCT); Additional inotrope requirement (RCTs); Duration of inotropes (hours) (RCTs)

ASSESSMENT

**Problem**<br>Is the problem a priority?		
**JUDGEMENT**	**RESEARCH EVIDENCE**	**ADDITIONAL CONSIDERATIONS**
○ No<br>○ Probably no<br>○ Probably yes<br>● Yes<br>○ Varies<br>○ Don't know	Neonatal shock is associated with significant morbidity and mortality. The use of hydrocortisone (HC) as an adjunct to inotropes in the treatment of neonatal shock is on the rise, and more so in extremely low birth weight (ELBW) neonates. (1) HC administration in vasopressor resistant shock has been shown to be associated with improved blood pressure (BP) eventually enabling the reduction of inotrope dosage. (2) There are no specific guidelines for HC usage in vasopressor resistant shock in neonates, especially with relation to its timing of initiation and dosage.	There is a lacunae in the literature as to when HC has to be considered in neonates with fluid refractory shock. While early administration may decrease morbidities secondary to tissue hypoperfusion, it may also result in its overuse which itself could be associated with short-term adverse effects such as spontaneous intestinal perforation (SIP), hyperglycemia and hypertension. (3, 4) On the other hand, late initiation of HC might prolong inotrope therapy, and hence increase the side effects secondary to its use. Hence, this systematic review and meta-analysis was conducted specifically to evaluate the timing of HC initiation in relation to inotrope therapy.
**Desirable Effects**<br>How substantial are the desirable anticipated effects?		
**JUDGEMENT**	**RESEARCH EVIDENCE**	**ADDITIONAL CONSIDERATIONS**
○ Trivial<br>○ Small<br>● Moderate<br>○ Large<br>○ Varies	The response to treatment (an important outcome) was significantly higher in the early HC group when compared to no HC group **[Risk ratio (RR): 1.85, 95% CI: 1.26-2.71); Risk difference (RD): 376 more per 1,000 (from 115 more to 756 more)].**<br>Early HC therapy possibly decreased the risk of requirement of additional inotropes	Heckman et al. in their retrospective study of 30 preterm neonates with shock had shown that **when HC was initiated in infants with shock prior to vasopressor therapy, 16 out of 30 neonates did not require vasopressor therapy.** (5)

○ Don't know	**(RR: 0.18, 95% CI: 0.05-0.69); RD: 281 fewer per 1,000 (from 326 fewer to 106 fewer).**<br>Early HC addition possibly decreased the duration of inotrope therapy as well **(MD: -39.8, 95% CI: -30.29 to -49.31**).<br>Administration of HC in addition to vasopressor therapy early possibly increased the mean BP **(MD: 7.3 mm Hg higher (4.4 higher to 10.2 higher).**<br>**Clinical benefit or harm could not be ruled out for the other critical and important outcomes that were synthesized in the meta-analysis.**	Kovacs et al. in their RCT enrolled term neonates undergoing therapeutic hypothermia who were diagnosed with shock and compared early HC with inotropes vs. inotropes alone. (6) **The cumulative and maximum dose of inotrope therapy was significantly lesser in HC group. The regression model predicted that early HC was associated with a 2.2 mm Hg increase in pulse pressure.**
**Undesirable Effects**<br>How substantial are the undesirable anticipated effects?		
**JUDGEMENT**	**RESEARCH EVIDENCE**	**ADDITIONAL CONSIDERATIONS**
● Trivial<br>○ Small<br>○ Moderate<br>○ Large<br>○ Varies<br>○ Don't know	Ng et al. found a **higher incidence of glycosuria** in the early HC group (p=0.03). (2)	Bourchier et al. in in their RCT had compared HC prior to inotrope use vs. inotrope alone. This study **found no statistically significant differences in the critical and important outcomes of survival, BPD, ROP (stage 2-4), IVH (grades 2-4), NEC, symptomatic PDA and sepsis.** (7)<br>Kovacs et al. in their RCT reported that early HC along with inotropes **decreased the risk of electrographic seizures**, but the requirement of anti-epileptic drug therapy and abnormal MRI was similar in both the groups.(6)<br>**Though, the research evidence did not show an increased risk of adverse events such as hyperglycemia or spontaneous intestinal perforation (SIP), HC use in conjunction with indomethacin has been shown to be associated with an increased risk of SIP.** (3)<br>Further, no study had reported on the long-term neurodevelopmental outcomes.
**Certainty of evidence**<br>What is the overall certainty of the evidence of effects?		
**JUDGEMENT**	**RESEARCH EVIDENCE**	**ADDITIONAL CONSIDERATIONS**

● Very low<br>○ Low<br>○ Moderate<br>○ High<br>○ No included studies	The Certainty of evidence was very low to low (predominantly very low) for all the outcomes evaluated.	
**Values**<br>Is there important uncertainty about or variability in how much people value the main outcomes?		
**JUDGEMENT**	**RESEARCH EVIDENCE**	**ADDITIONAL CONSIDERATIONS**
○ Important uncertainty or variability<br>○ Possibly important uncertainty or variability<br>● Probably no important uncertainty or variability<br>○ No important uncertainty or variability	No studies were found.	Our judgement is that there is probably no important uncertainty regarding the critical and important outcomes that were evaluated. This is based on the consensus of the working group.
**Balance of effects**<br>Does the balance between desirable and undesirable effects favor the intervention or the comparison?		
**JUDGEMENT**	**RESEARCH EVIDENCE**	**ADDITIONAL CONSIDERATIONS**
○ Favors the comparison<br>○ Probably favors the comparison<br>○ Does not favor either the intervention or the comparison<br>● Probably favors the intervention<br>○ Favors the intervention<br>○ Varies<br>○ Don't know	The **response to treatment (an important outcome) was significantly higher in the early HC group when compared to no HC group [Risk ratio (RR): 1.85, 95% CI: 1.26-2.71); Risk difference (RD): 376 more per 1,000 (from 115 more to 756 more)].** Early HC therapy **possibly decreased the risk of requirement of additional inotropes [RR: 0.18, 95% CI: 0.05-0.69); RD: 281 fewer per 1,000 (from 326 fewer to 106 fewer)].**Early HC addition **possibly decreased the duration of inotrope therapy (MD: -39.8, 95% CI: -30.29 to -49.31).**<br>Administration of HC in addition to vasopressor therapy early **possibly increased the mean BP (MD: 7.3 mm Hg higher (4.4 higher to 10.2 higher).** Clinical benefit or harm could not be ruled out for the other critical and important outcomes synthesized in the meta-analysis.<br>The only adverse event reported was by Ng et al. who found **a higher incidence of glycosuria in the early HC group** (p=0.03). (2)<br>Considering all these results, we adjudged that the balance of effects probably favours the intervention which is early HC along with inotrope therapy.	Bourchier et al. in in their RCT had compared HC prior to inotrope use vs. inotrope alone. This study which had used relatively higher doses of HC did not find any **statistically significant differences in the critical and important outcomes of survival, BPD, ROP (stage 2-4), IVH (grades 2-4), NEC, symptomatic PDA and sepsis.** (7)<br>Heckman et al. in their retrospective study of 30 preterm neonates with shock had shown that **when HC was initiated in infants with shock prior to vasopressor therapy, 16 out of 30 neonates did not require vasopressor therapy with no adverse events.** (5)<br>Kovacs et al. in their RCT enrolled term neonates undergoing therapeutic hypothermia who were diagnosed with shock and compared early HC with inotropes vs. inotropes alone. (6) The authors reported that **HC group had statistically**

		**significant decreased risk of electrographic seizures, but the requirement of anti-epileptic drug therapy and abnormal MRI was similar in both the groups. Also, the cumulative and maximum dose of inotrope therapy was significantly lesser in HC group. The regression model predicted that early HC was associated with a 2.2 mm Hg increase in pulse pressure.** (6) Though, the research evidence did not show an increased risk of adverse events such as hyperglycemia or spontaneous intestinal perforation (SIP), **HC use in conjunction with indomethacin has been shown to be associated with an increased risk of SIP**.(3) **Further, the studies included in the meta-analysis were underpowered to detect significant differences in most of the critical and important outcomes. Finally, none of the included studies had reported on the long-term outcomes such as neurodevelopmental impairment.**
**Resources required**<br>How large are the resource requirements (costs)?"		
**JUDGEMENT**	**RESEARCH EVIDENCE**	**ADDITIONAL CONSIDERATIONS**
○ Large costs<br>○ Moderate costs<br>● Negligible costs and savings<br>○ Moderate savings<br>○ Large savings<br>○ Varies<br>○ Don't know	No study had evaluated the resources required.	HC is a low cost drug with easy accessibility. The other resources required such as syringes, equipment for BP monitoring and blood glucose monitoring are routinely utilized in NICUs.
**Certainty of evidence of required resources**<br>What is the certainty of the evidence of resource requirements (costs)?		
**JUDGEMENT**	**RESEARCH EVIDENCE**	**ADDITIONAL CONSIDERATIONS**

○ Very low<br>○ Low<br>○ Moderate<br>○ High<br>● No included studies	No study had evaluated the resources required.	
**Cost effectiveness**<br>Does the cost-effectiveness of the intervention favor the intervention or the comparison?		
**JUDGEMENT**	**RESEARCH EVIDENCE**	**ADDITIONAL CONSIDERATIONS**
○ Favors the comparison<br>○ Probably favors the comparison<br>○ Does not favor either the intervention or the comparison<br>● Probably favors the intervention<br>○ Favors the intervention<br>○ Varies<br>○ No included studies	No studies were identified.	Early HC by decreasing the duration of inotrope use, reducing the requirement of additional inotropes and by improving the response to therapy with no possible adverse events might be more cost-effective than the comparison group. This was based on consensus of the working group.
**Equity**<br>What would be the impact on health equity?		
**JUDGEMENT**	**RESEARCH EVIDENCE**	**ADDITIONAL CONSIDERATIONS**
○ Reduced<br>○ Probably reduced<br>○ Probably no impact<br>● Probably increased<br>○ Increased<br>○ Varies<br>○ Don't know		Early HC by decreasing the inotrope duration, requirement of additional inotropes and by improving the response to therapy might improve equity by allowing for the available manpower and equipment to be utilized for other sicker neonates who require treatment with infusion pumps and monitoring using equipment such as multi-para monitors. This was based on the consensus of the working group.
**Acceptability**<br>Is the intervention acceptable to key stakeholders?		

JUDGEMENT	RESEARCH EVIDENCE	ADDITIONAL CONSIDERATIONS
○ No<br>○ Probably no<br>○ Probably yes<br>○ Yes<br>○ Varies<br>● Don't know	No studies were identified.	Though the research evidence did not show any major harm with early HC, its use has been reported to be associated with SIP. This may or may not influence the acceptability for its early use in neonates with fluid refractory shock. This was based on the consensus of the working group.

Feasibility
Is the intervention feasible to implement?

JUDGEMENT	RESEARCH EVIDENCE	ADDITIONAL CONSIDERATIONS
○ No<br>○ Probably no<br>● Probably yes<br>○ Yes<br>○ Varies<br>○ Don't know	No studies were identified.	HC is an easily accessible drug and does not require any major equipment for its use.

SUMMARY OF JUDGEMENTS

	JUDGEMENT						
**PROBLEM**	No	Probably no	Probably yes	**Yes**		Varies	Don't know
**DESIRABLE EFFECTS**	Trivial	Small	**Moderate**	Large		Varies	Don't know
**UNDESIRABLE EFFECTS**	**Trivial**	Small	Moderate	Large		Varies	Don't know
**CERTAINTY OF EVIDENCE**	**Very low**	Low	Moderate	High			No included studies
**VALUES**	Important uncertainty or variability	Possibly important uncertainty or variability	**Probably no important uncertainty or variability**	No important uncertainty or variability			
**BALANCE OF EFFECTS**	Favors the comparison	Probably favors the comparison	Does not favor either the intervention or the comparison	**Probably favors the intervention**	Favors the intervention	Varies	Don't know
**RESOURCES REQUIRED**	Large costs	Moderate costs	**Negligible costs and savings**	Moderate savings	Large savings	Varies	Don't know

	JUDGEMENT						
**CERTAINTY OF EVIDENCE OF REQUIRED RESOURCES**	Very low	Low	Moderate	High			**No included studies**
**COST EFFECTIVENESS**	Favors the comparison	Probably favors the comparison	Does not favor either the intervention or the comparison	**Probably favors the intervention**	Favors the intervention	Varies	No included studies
**EQUITY**	Reduced	Probably reduced	Probably no impact	**Probably increased**	Increased	Varies	Don't know
**ACCEPTABILITY**	No	Probably no	Probably yes	Yes		Varies	**Don't know**
**FEASIBILITY**	No	Probably no	**Probably yes**	Yes		Varies	Don't know

TYPE OF RECOMMENDATION

Strong recommendation against the intervention	Conditional recommendation against the intervention	Conditional recommendation for either the intervention or the comparison	**Conditional recommendation for the intervention**	Strong recommendation for the intervention
○	○	○	●	○

CONCLUSIONS

Recommendation

Early hydrocortisone (HC) may be used in the treatment of neonates with fluid refractory shock as an adjunct to the first line inotrope in neonates. There is insufficient evidence to adjudge the most appropriate timing of its use in relation to the dose of the first inotrope. We suggest to initiate HC therapy with increasing requirement of the first inotrope. The most commonly used first line inotrope in the studies evaluated was dopamine, and HC therapy may be initiated if the neonate requires dopamine ≥ 10 mcg/kg/min. There is insufficient evidence to recommend the timing of introduction of HC in relation to other inotropes when used as first line such as epinephrine, norepinephrine, vasopressin and milrinone. In such scenarios, the guideline panel suggests using dopamine equivalent doses, which adjudged according to the vasoactive inotropic score are as follows: dobutamine (≥ 10 mcg/kg/min), epinephrine (≥ 0.1 mcg/kg/min), norepinephrine (≥ 0.1 mcg/kg/min), vasopressin (≥0.0001 IU/Kg/min) and milrinone (l mcg/kg/min) (weak recommendation, very low certainty of evidence).

The dosage of HC utilized in the studies was widely varied. We suggest the following HC dosage:

- 1 mg/kg followed by 0.5-1 mg/kg every 8-12 hourly may be considered in preterm infants.
- 2 mg/kg followed by 1 mg/kg every 6-8 hourly may be considered in term neonates.

HC may be tapered over 2-3 days once the desired effect has been achieved which is when the neonate is being weaned from inotropes.

Justification

The use of early HC was adjudged to have moderate substantial effects. Further, there were trivial undesirable effects. The balance of effects also favoured early HC therapy. Though no studies had evaluated its cost effectiveness and the resources required, our judgement is that they may be favourable towards early HC therapy. Similarly, the parameters of equity and feasibility might favour early HC therapy. However, the certainty of evidence for the outcomes evaluated was very low, and most of the studies were underpowered to detect critical and important outcomes. Low dose HC prophylaxis has become a standard of care in extremely low gestational age neonates especially those born through chorioamnionitis. All of the aforementioned parameters were considered before making the recommendation.

Subgroup considerations

There was insufficient data to perform any sub-group analyses.

Implementation considerations

- HC should not be used concurrently with indomethacin.
- Blood sugars should be monitored while the neonate is treated with HC.
- Vasoactive inotropic score (VAI) = 1 x dopamine (mcg/kg/min) + 1 x dobutamine (mcg/kg/min) + 10 x milrinone (mcg/kg/min) + 100 x epinephrine (mcg/kg/min) + 100 x norepinephrine (mcg/kg/min) + 10000 x vasopressin (IU/kg/min).

Research priorities

- There are no RCTs comparing the timing of HC initiation in relation to inotrope therapy in neonates with shock and further research is warranted. Future research may also focus on the use of HC when the first line inotrope of choice is dobutamine or epinephrine or norepinephrine or vasopressin or milrinone.
- The use of cortisol levels to initiate and monitor HC therapy has been studied but with contentious results. Hence, further research is warranted with respect to this. (6, 8, 9, 10, 7, 11)
- The safety of use of HC along with paracetamol (an alternative to NSAIDs) for the treatment of PDA which is used widely nowadays requires further studies.

REFERENCES SUMMARY

1. Rios DR, Moffett BS, Kaiser JR. Trends in pharmacotherapy for neonatal hypotension. J Pediatr. 2014 ;165(4):697-701.e1.
2. Ng PC, Lee CH, Bnur FL, Chan IH, Lee AW, Wong E, et al. A double-blind, randomized, controlled study of a “stress dose” of hydrocortisone for rescue treatment of refractory hypotension in preterm infants. Pediatrics. 2006;117(2):367-75.
3. Ramaswamy VV, Bandyopadhyay T, Nanda D, Bandiya P, Ahmed J, Garg A, et al.; Assessment of Postnatal Corticosteroids for the Prevention of Bronchopulmonary Dysplasia in Preterm Neonates: A Systematic Review and Network Meta-analysis. JAMA Pediatr. 2021;175(6):e206826.
4. Kumbhat N, Noori S. Corticosteroids for Neonatal Hypotension. Clin Perinatol. 2020 ;47(3):549-562.
5. Heckmann M, Pohlandt F. Hydrocortisone in preterm infants. Pediatrics. 2002 ;109(6):1184-5; author reply 1184-5.
6. Kovacs K, Szakmar E, Meder U, Szakacs L, Cseko A, Vatai B, et al. A Randomized Controlled Study of Low-Dose Hydrocortisone Versus Placebo in Dopamine-Treated Hypotensive Neonates Undergoing Hypothermia Treatment for Hypoxic-Ischemic Encephalopathy. J Pediatr. 2019;211:13-19.e3.
7. Bourchier D, Weston PJ. Randomised trial of dopamine compared with hydrocortisone for the treatment of hypotensive very low birthweight infants. Arch Dis Child Fetal Neonatal Ed. 1997 ;76(3):F174-8.
8. Noori S, Friedlich P, Wong P, Ebrahimi M, Siassi B, Seri I. Hemodynamic changes after low-dosage hydrocortisone administration in vasopressor-treated preterm and term neonates. Pediatrics. 2006 ;118(4):1456-66.
9. Peeples ES. An evaluation of hydrocortisone dosing for neonatal refractory hypotension. J Perinatol. 2017 ;37(8):943-946.
10. Ramanathan R, Siassi B, Sardesai S. DEXAMETHASONE VERSUS HYDROCORTISONE FOR HYPOTENSION REFRACTORY TO HIGH DOSE INOTROPIC AGENTS AND INCIDENCE OF CANDIDA INFECTION IN EXTREMELY LOW BIRTH WEIGHT INFANTS. Pediatr Res.1996;39 (Suppl 4):240.
11. Robertson JO, Criss CN, Hsieh LB, Matsuko N, Gish JS, Mon RA et al. Steroid use for refractory hypotension in congenital diaphragmatic hernia. Pediatr Surg Int. 2017;33(9):981-987.

Supplement Table 7: EtD framework forlate hydrocortisone

Should late hydrocortisone (when a high dosage of the first inotrope is reached or when additional inotropes are required along with the first inotrope) vs. inotropes alone be used for neonates with shock?	
**POPULATION:**	Neonates with fluid refractory (as defined by the authors) shock.
**INTERVENTION:**	Late hydrocortisone (when a high dosage of the first inotrope is reached or when additional inotropes are required along with the first inotrope). A vasoactive inotropic score of > 10 was used to define "high dosage of the first inotrope".
**COMPARISON:**	Inotropes alone
**MAIN OUTCOMES:**	Mortality until discharge (RCT); Mortality until discharge (non-RCTs); Culture positive sepsis (RCT); Culture positive sepsis (non-RCT); NEC ≥ stage 2 (non-RCT); Response to treatment (RCT); Change in BP (non-RCT); Duration of inotropes (hours) (non-RCT); Duration of ventilation (hours) (non-RCTs); Duration of hospitalisation (days) (non-RCT).

ASSESSMENT

**Problem**<br>Is the problem a priority?		
**JUDGEMENT**	**RESEARCH EVIDENCE**	**ADDITIONAL CONSIDERATIONS**
○ No<br>○ Probably no<br>○ Probably yes<br>● Yes<br>○ Varies<br>○ Don't know	Neonatal shock is associated with significant morbidity and mortality. The use of hydrocortisone (HC) as an adjunct to inotropes in the treatment of neonatal shock is on the rise, and more so in extremely low birth weight (ELBW) neonates. (1) HC administration in vasopressor resistant shock has been shown to be associated with improved blood pressure (BP) eventually enabling the reduction of inotrope dosage. (2) There are no specific guidelines for HC usage in vasopressor resistant shock in neonates, especially with relation to its timing of initiation and dosage.	There is a lacunae in the literature as to when HC has to be considered in neonates with fluid refractory shock. While early administration may decrease morbidities secondary to tissue hypoperfusion, it may also result in its overuse which itself could be associated with short-term adverse effects such as spontaneous intestinal perforation (SIP), hyperglycemia and hypertension. (3, 4) On the other hand, late initiation of HC might prolong inotrope therapy, and hence increase the side effects secondary to its use. Hence, this systematic review and meta-analysis was conducted specifically to evaluate the timing of HC initiation in relation to inotrope therapy.
**Desirable Effects**<br>How substantial are the desirable anticipated effects?		
**JUDGEMENT**	**RESEARCH EVIDENCE**	**ADDITIONAL CONSIDERATIONS**
○ Trivial<br>● Small<br>○ Moderate<br>○ Large<br>○ Varies<br>○ Don't know	Late HC therapy in addition to inotropes possibly **increased the response to treatment (important outcome) [Risk ratio (RR): 2.50 (1.16 to 5.39); Risk difference (RD): 360 more per 1,000 (from 38 more to – (upper limit of 95% CI not calculable)].** (5) **Since the lower limit of 95% confidence interval (CI) for the RD was 3.8 %, we classified the magnitude of effects as being small.**<br>Late HC also possibly increased the mean BP **[(MD: 10.78 mm Hg higher (8.59**	Baker et al. in their retrospective study also had reported that late HC (when highest dosages of dopamine and dobutamine were reached) **increased the MAP at 2, 6, 12 and 24 hours after initiation, decreased the total inotrope dose at 6, 12 and 24 hours, and was associated with resolution of**

	**higher to 12.98 higher)].** (6, 7, 8)	**oliguria.** (9)<br>Helbock et al. in their prospective study reported an **increase in BP as early as one-half hours following the administration of late HC (HC added after highest dosages of vasopressor therapy was reached), and that within 12 to 30 hours all the newborns were normotensive without inotrope support.** (10)<br>Krediet et al. in their RCT reported **no difference in additional requirement of inotropes or volume expanders in the late HC group (HC was initiated if the neonate was hypotensive despite receiving dopamine and / or dobutamine at 20 ug/kg/min). However, in the late HC group the increase in BP to normal values occurred more rapidly (within 6 h).** (11)<br>Vishveshwara et al. in their pre-post study indicated that late HC (HC was initiated when the requirement of Dopamine was >17 ug/kg/min) **was significantly efficacious in reducing the dose of dopamine and in maintaining the BP within the target limits.** (12)

Undesirable Effects
How substantial are the undesirable anticipated effects?

JUDGEMENT	RESEARCH EVIDENCE	ADDITIONAL CONSIDERATIONS
○ Trivial<br>○ Small<br>● Moderate<br>○ Large<br>○ Varies<br>○ Don't know	**Clinical benefit or harm could not be ruled out for the critical outcome of mortality and the important outcome of culture positive sepsis from the data synthesis which included only 1 RCT and 2 observational studies.**<br>**The risk of the important outcome of NEC ≥ stage 2 was higher in the late HC group [RR: 2.46 (1.19 to 5.08); RD: 381 more per 1,000 (from 50 more to 1,000 more)]. The duration of inotrope therapy was also possibly higher in the late HC group [MD 61.75 hours higher (43.98 higher to 79.52 higher)]. Further, the duration of hospital stay was possibly higher in the late HC group [MD: 38 days higher (12.11 higher to 63.89 higher)].** These outcomes were reported by a single observational study. (8) **Though the gestational age was slightly lower in the late HC group** when compared to the inotrope only group in this study **(26 weeks vs. 27.1 weeks), all other baseline parameters were similar.** (8) The mean vasoactive index (VAI) score in the late HC group was 15. The study also reported that **after adjustment for baseline sickness, multi-variate analysis showed that receipt of late HC and gestational age were independently associated with higher risk of death and / or BPD at 36 weeks' PMA. Finally, after adjustment for baseline sickness, the late HC group was shown to have higher risk of mortality at 1 year PMA as well (Hazard Ratio: 6.08, P=0.01).**	Noori et al. in their prospective observational study reported that neonates treated with late HC along with inotropes (HC was started at ≥ 15 ug/kg/min of dopamine and / or dobutamine) and with **a pre-treatment cortisol level of >15 mcg/dl had significantly higher rates of hyperglycemia, requirement of insulin therapy and death than those with levels <15 mcg/dl.** (7)<br>Peeples et al. in their retrospective cohort study evaluated the effect of late HC (mean vasopressor dose of 12.4 ug/kg/min) in preterm infants. (13) The authors found that the late HC group had **higher incidence of hypertension (p=0.02) and hyperglycemia (p=0.02).** When a high dosage (4 mg/kg/d) vs. low dosage (1-3 mg/kg/d) of HC was compared, **the high dose group had significantly higher incidence of hypertension (p=0.02).**<br>Further, those **with pre-treatment cortisol level of >15 ug/dl had higher rates of hyperglycemia**

		**(p=0.01), insulin therapy (p=0.03) and death (p=0.001)** when compared to those with ≤15 ug/dl. **The increased incidence of death in high cortisol group was independent of the HC dose used.** Verma et al. in their retrospective cohort study reported that **ELBW infants of ≤25 w of GA, unexposed to ANS and born to GDM mothers were at significantly higher risk of refractory hypotension** [Refractory hypotension was defined as hypotension persisting despite treatment with maximum doses of dopamine / dobutamine (20 ug/kg/min) and/or epinephrine(0.5–1 ug/kg/min) in addition to volume expansion)]. **No difference in BPD, PDA, NEC and SIP was found between the 2 groups. The authors concluded that such neonates may benefit from an initial therapy with, or earlier institution of hydrocortisone.** (14) Ramanathan et al. in their retrospective study reported that **9 out of 19 neonates who received late HC therapy (when highest dosages of dopamine and dobutamine were reached) developed fungal sepsis. No difference in death and incidence of bacterial sepsis was noted between the groups.** (15) Krediet et al. **did not find any difference between the two groups for the outcome of any IVH.** (11) Baker et al. also reported **no difference in IVH > grade 2, PVL, bacterial or fungal sepsis and spontaneous intestinal perforation with late HC treatment.** (9)

Certainty of evidence
What is the overall certainty of the evidence of effects?

JUDGEMENT	RESEARCH EVIDENCE	ADDITIONAL CONSIDERATIONS
● Very low<br>○ Low<br>○ Moderate<br>○ High<br>○ No included studies	The Certainty of evidence was very low to low (predominantly very low) for all the outcomes evaluated.	

**Values**<br>Is there important uncertainty about or variability in how much people value the main outcomes?		
**JUDGEMENT**	**RESEARCH EVIDENCE**	**ADDITIONAL CONSIDERATIONS**
○ Important uncertainty or variability<br>○ Possibly important uncertainty or variability<br>● Probably no important uncertainty or variability<br>○ No important uncertainty or variability	No studies were found.	Our judgement is that there is probably no important uncertainty regarding the critical and important outcomes that were evaluated. This is based on the consensus of the working group.

**Balance of effects**<br>Does the balance between desirable and undesirable effects favor the intervention or the comparison?		
**JUDGEMENT**	**RESEARCH EVIDENCE**	**ADDITIONAL CONSIDERATIONS**
○ Favors the comparison<br>● Probably favors the comparison<br>○ Does not favor either the intervention or the comparison<br>○ Probably favors the intervention<br>○ Favors the intervention<br>○ Varies<br>○ Don't know	Though only 1 RCT and 2 observational studies were included in the summary of findings (SoF) table, the results for benefit vs. harm was also evaluated from many observational studies and RCTs that could not be pooled in a meta-analysis. Apart from **the increased risk of NEC ≥ stage 2, the increased duration of inotrope requirement and the duration of hospital stay with late HC as indicated by the studies included in the SoF table, most of the other studies that could not be evaluated through the GRADE process had also indicated the possibility of significant harm with late HC including increased risk of mortality, fungal sepsis, hypertension and hyperglycemia requiring insulin therapy**. **There were a few studies which had reported the possibility of no increase in the risk of mortality, SIP, severe IVH, BPD and PDA as well. However, these were outweighed in number and quality of the other studies which had suggesting the possibility of harm. Hence, we adjudged that the balance of effects probably favours the comparison.**	

**Resources required**<br>How large are the resource requirements (costs)?"		
**JUDGEMENT**	**RESEARCH EVIDENCE**	**ADDITIONAL CONSIDERATIONS**
○ Large costs<br>○ Moderate costs<br>● Negligible costs and savings<br>○ Moderate savings<br>○ Large savings<br>○ Varies<br>○ Don't know	No study had evaluated the resources required.	HC is a low cost drug with easy accessibility. The other resources required such as syringes, BP monitoring and blood glucose monitoring are routinely utilized in NICUs.

**Certainty of evidence of required resources**<br>What is the certainty of the evidence of resource requirements (costs)?		
**JUDGEMENT**	**RESEARCH EVIDENCE**	**ADDITIONAL CONSIDERATIONS**
○ Very low<br>○ Low<br>○ Moderate<br>○ High<br>● No included studies	No study had evaluated the resources required.	

**Cost effectiveness**<br>Does the cost-effectiveness of the intervention favor the intervention or the comparison?		
**JUDGEMENT**	**RESEARCH EVIDENCE**	**ADDITIONAL CONSIDERATIONS**
○ Favors the comparison<br>● Probably favors the comparison<br>○ Does not favor either the intervention or the comparison<br>○ Probably favors the intervention<br>○ Favors the intervention<br>○ Varies<br>○ No included studies	No studies were identified.	Late HC was associated with increased risk of adverse events as reported by most of the included studies. The consensus of the working group is that cost-effectiveness probably favours the comparison.

**Equity**<br>What would be the impact on health equity?		
**JUDGEMENT**	**RESEARCH EVIDENCE**	**ADDITIONAL CONSIDERATIONS**
○ Reduced<br>● Probably reduced<br>○ Probably no impact<br>○ Probably increased<br>○ Increased<br>○ Varies<br>○ Don't know		Our judgement based on consensus is that late HC by increasing the risk of adverse events might divert resources such as manpower and equipment which could be utilized for other sick neonates.

**Acceptability**<br>Is the intervention acceptable to key stakeholders?		
**JUDGEMENT**	**RESEARCH EVIDENCE**	**ADDITIONAL CONSIDERATIONS**
○ No<br>● Probably no<br>○ Probably yes<br>○ Yes<br>○ Varies<br>○ Don't know		Our judgement is that the key stakeholders including the treating clinicians, parents and policy makers probably might not accept the intervention (late HC). Our judgement is based on the parameter of balance of effects which has been explained in detail in the respective section

**Feasibility**<br>Is the intervention feasible to implement?		
**JUDGEMENT**	**RESEARCH EVIDENCE**	**ADDITIONAL CONSIDERATIONS**
○ No<br>○ Probably no<br>● Probably yes<br>○ Yes<br>○ Varies<br>○ Don't know	No studies were identified.	HC is an easily accessible drug and does not require any major equipment for its use.

SUMMARY OF JUDGEMENTS

	**JUDGEMENT**						
**PROBLEM**	No	Probably no	Probably yes	**Yes**		Varies	Don't know
**DESIRABLE EFFECTS**	Trivial	**Small**	Moderate	Large		Varies	Don't know
**UNDESIRABLE EFFECTS**	Trivial	Small	**Moderate**	Large		Varies	Don't know
**CERTAINTY OF EVIDENCE**	**Very low**	Low	Moderate	High			No included studies
**VALUES**	Important uncertainty or variability	Possibly important uncertainty or variability	**Probably no important uncertainty or variability**	No important uncertainty or variability			
**BALANCE OF EFFECTS**	Favors the comparison	**Probably favors the comparison**	Does not favor either the intervention or the comparison	Probably favors the intervention	Favors the intervention	Varies	Don't know

	JUDGEMENT						
RESOURCES REQUIRED	Large costs	Moderate costs	**Negligible costs and savings**	Moderate savings	Large savings	Varies	Don't know
CERTAINTY OF EVIDENCE OF REQUIRED RESOURCES	Very low	Low	Moderate	High			**No included studies**
COST EFFECTIVENESS	Favors the comparison	**Probably favors the comparison**	Does not favor either the intervention or the comparison	Probably favors the intervention	Favors the intervention	Varies	No included studies
EQUITY	Reduced	**Probably reduced**	Probably no impact	Probably increased	Increased	Varies	Don't know
ACCEPTABILITY	No	**Probably no**	Probably yes	Yes		Varies	Don't know
FEASIBILITY	No	Probably no	**Probably yes**	Yes		Varies	Don't know

TYPE OF RECOMMENDATION

Strong recommendation against the intervention	Conditional recommendation against the intervention	Conditional recommendation for either the intervention or the comparison	Conditional recommendation for the intervention	Strong recommendation for the intervention
○	●	○	○	○

CONCLUSIONS

Recommendation

The guideline panel suggests that late hydrocortisone therapy as an adjunct to inotropes may not be used in the treatment of neonates with fluid refractory shock. (weak recommendation, very low certainty of evidence). This recommendation is to be interpreted along with the recommendation for earlier use of hydrocortisone along with inotropes in neonates with fluid refractory shock.

Justification

The use of late HC along with inotropes was adjudged to have significant side effects including mortality, NEC ≥ stage 2, fungal sepsis, hypertension, hyperglycemia requiring insulin therapy., increased duration of inotropes as well as hospital stay. Further, there were small desirable effects such as response to therapy and improved BP. Hence, the balance of effects favoured the comparator group of inotrope therapy alone. Though no studies had evaluated its cost effectiveness and the resources required, our judgement is that they may be not be favourable towards the intervention group of late HC along with inotropes. Similarly, the parameters of equity and values also favoured the comparator group of inotrope alone. All of the aforementioned parameters were considered before making this recommendation. The recommendation for earlier HC was also taken into account while making this recommendation.

Subgroup considerations

There was insufficient data to perform any sub-group analyses.

Implementation considerations

None

Research priorities

- There are no RCTs evaluating the timing of HC initiation in neonates with shock and research related to this PICO is warranted.
- The use of cortisol levels to initiate and monitor HC therapy has been studied but with contentious results. Hence further research is warranted with respect to this as well. (16, 7, 13, 15, 17, 18)

REFERENCES SUMMARY

1. Rios DR, Moffett BS, Kaiser JR. Trends in pharmacotherapy for neonatal hypotension. J Pediatr. 2014 ;165(4):697-701.e1.
2. Ng PC, Lee CH, Bnur FL, Chan IH, Lee AW, Wong E, et al. A double-blind, randomized, controlled study of a “stress dose” of hydrocortisone for rescue treatment of refractory hypotension in preterm infants. Pediatrics. 2006;117(2):367-75.
3. Ramaswamy VV, Bandyopadhyay T, Nanda D, Bandiya P, Ahmed J, Garg A, et al. Assessment of Postnatal Corticosteroids for the Prevention of Bronchopulmonary Dysplasia in Preterm Neonates: A Systematic Review and Network Meta-analysis. JAMA Pediatr. 2021;175(6):e206826.
4. Kumbhat N, Noori S. Corticosteroids for Neonatal Hypotension. Clin Perinatol. 2020 ;47(3):549-562.
5. Salas G, Travaglianti M, Leone A, Couceiro C, Rodríguez S. Hydrocortisone for the treatment of refractory hypotension: a randomized controlled trial. An Pediatr (Barc). 2014;80(6):387-93.
6. Seri I. Hydrocortisone and vasopressor-resistant shock in preterm neonates. Pediatrics. 2006;117(2):516-8.
7. Noori S, Friedlich P, Wong P, Ebrahimi M, Siassi B, Seri I. Hemodynamic changes after low-dosage hydrocortisone administration in vasopressor-treated preterm and term neonates. Pediatrics. 2006 ;118(4):1456-66.
8. Altit G, Vigny-Pau M, Barrington K, Dorval VG, Lapointe A. Corticosteroid Therapy in Neonatal Septic Shock-Do We Prevent Death? Am J Perinatol. 2018 ;35(2):146-151.
9. Baker CF, Barks JD, Engmann C, Vazquez DM, Neal CR Jr, Schumacher RE, Bhatt-Mehta V. Hydrocortisone administration for the treatment of refractory hypotension in critically ill newborns. J Perinatol. 2008 ;28(6):412-9.
10. Helbock HJ, Insoft RM, Conte FA. Glucocorticoid-responsive hypotension in extremely low birth weight newborns. Pediatrics. 1993;92(5):715-7.
11. Krediet GT, van der Ent K, Rademaker MAK, van Bel F. Rapid Increase of Blood Pressure after Low Dose Hydrocortison (HC) in Low Birth Weight Neonates with Hypotension Refractory to High Doses of Cardio-inotropics. Pediatr Res. 1998; 43:38.
12. Visveshwara N, Peck M, Wells R, Bansal V, Chopra D, Rajani K. Efficacy of hydrocortisone in restoring blood pressure in infants on dopamine therapy. Pediatr Res. 1996; 39:251.
13. Peeples ES. An evaluation of hydrocortisone dosing for neonatal refractory hypotension. J Perinatol. 2017 ;37(8):943-946.
14. Verma RP, Dasnadi S, Zhao Y, Chen HH. A comparative analysis of ante- and postnatal clinical characteristics of extremely premature neonates suffering from refractory and non-refractory hypotension: Is early clinical differentiation possible? Early Hum Dev. 2017 ;113:49-54.
15. Ramanathan R, Siassi B, Sardesai S. Dexamethasone Versus Hydrocortisone For Hypotension Refractory To High Dose Inotropic Agents And Incidence Of Candida Infection In Extremely Low Birth Weight Infants. Pediatr Res.1996;39 (Suppl 4):240.
16. Kovacs K, Szakmar E, Meder U, Szakacs L, Cseko A, Vatai B et al. A Randomized Controlled Study of Low-Dose Hydrocortisone Versus Placebo in Dopamine-Treated Hypotensive Neonates Undergoing Hypothermia Treatment for Hypoxic-Ischemic Encephalopathy. J Pediatr. 2019;211:13-19.e3.
17. Bourchier D, Weston PJ. Randomised trial of dopamine compared with hydrocortisone for the treatment of hypotensive very low birthweight infants. Arch Dis Child Fetal Neonatal Ed. 1997 ;76(3):F174-8.
18. Robertson JO, Criss CN, Hsieh LB, Matsuko N, Gish JS, Mon RA, Johnson KN, Gadepalli SK. Steroid use for refractory hypotension in congenital diaphragmatic hernia. Pediatr Surg Int. 2017;33(9):981-987.
